# The rise of serotype 8 is associated with lineages and mutations in the capsular operon with different potential to produce invasive pneumococcal disease

**DOI:** 10.1080/22221751.2025.2521845

**Published:** 2025-06-16

**Authors:** Covadonga Pérez-García, Aída González-Díaz, Mirian Domenech, Mirella Llamosí, Aída Úbeda, Juan Carlos Sanz, Ernesto García, Carmen Ardanuy, Julio Sempere, Jose Yuste

**Affiliations:** a Spanish Pneumococcal Reference Laboratory, National Center for Microbiology, Instituto de Salud Carlos III, Madrid, Spain; b Microbiology Department, Hospital Universitari de Bellvitge, Barcelona, Spain; c CIBER de Enfermedades Respiratorias (CIBERES), Instituto de Salud Carlos III, Madrid, Spain; d Regional Public Health Laboratory, Comunidad de Madrid, Madrid, Spain; e CIBER de Epidemiología y Salud Pública (CIBERESP), Instituto de Salud Carlos III, Madrid, Spain; f Centro de Investigaciones Biológicas Margarita Salas-CSIC, Madrid, Spain

**Keywords:** IPD, serotype 8, GPSC3, GPSC98, GPSC9, *Wcha*, mucoid colonies, *Pspc*

## Abstract

Despite conjugate vaccine introduction to prevent invasive pneumococcal disease (IPD), serotype replacement by non-vaccine serotypes is a constant concern. In this study, we elucidate the rise of serotype 8 causing IPD in Spain. We evaluated isolates received during the period 2008–2023 including whole genome sequencing characterization and host–pathogen interaction studies. Serotype 8 has emerged as one of the most prevalent serotypes causing IPD in both children and adults. CC53/GPSC3 carrying *pspC* 6.11 was the dominant lineage in recent years, displaying increased adhesion to lung cells, enhanced biofilm formation, higher factor H recruitment, improved phagocytosis evasion, and greater virulence in a mice pneumonia model than other co-circulating lineages which could explain its predominance. Morphologically, serotype 8 strains exhibit two appearances on blood agar plates: mucoid colonies, and non-mucoid variants. Molecular characterization revealed that non-mucoid variants harbour mutations in the *wchA* gene and/or others within the capsular operon, leading to increased adhesion and biofilm formation, albeit with reduced immune evasion capacity. Serotype 8 has become a major cause of IPD, with CC53/GPSC3 as the dominant lineage due to its pathogenic advantages. The versatility of the capsular operon contributes to its success in causing IPD. The use of vaccines with broader coverage, such as PCV20 or PCV21, containing this serotype, may offer an effective strategy to ameliorate the impact on IPD by serotype 8.

## GRAPHICAL ABSTRACT

**Figure:**
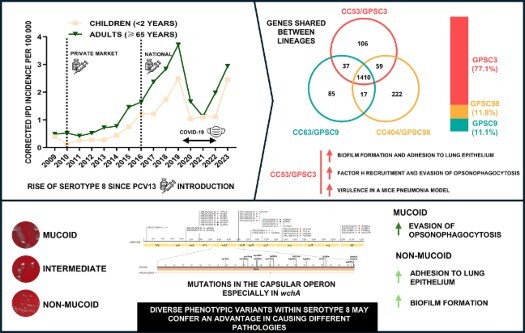

## Introduction

Lower respiratory tract infectious diseases (LRTID) are currently responsible for high morbidity and mortality rates, and even before the coronavirus disease 2019 (COVID-19) pandemic caused by severe acute respiratory syndrome coronavirus 2 (SARS-CoV-2), LRTID stood as one of the major causes of mortality worldwide [1]. With approximately 2.28 million deaths attributed to LRTID, *Streptococcus pneumoniae*, or pneumococcus, was identified as the primary etiological agent responsible for these cases, affecting predominantly children under 5 years old and adults over 60 [1]. Adhesion to the upper respiratory tract is *sine qua non* condition for establishing invasive pneumococcal disease (IPD) as well as non-invasive conditions. Pneumococcal conjugate vaccines (PCVs) have played a crucial role in the prevention of IPD. However, due to the high diversity of pneumococcus with up to 107 serotypes described so far, limitation in serotype coverage and replacement by non-PCV13 serotypes are the main disadvantages of PCVs. In Spain, replacement by non-PCV13 serotypes has been shown both in children and adults after PCV13 introduction in 2010, even by serotypes included in the 23-valent polysaccharide vaccine (PPV23) that has been scarcely used in adults. Among these emergent serotypes not included in PCV13, serotype 8 increased in children and adults before the COVID-19 pandemic and in the last years is still a leading cause of IPD [2]. In addition, a rise of other non-PCV13 serotypes such as 22F and 24F has been observed in children, comparing the period 2009–2023 [2,3]. In adults, serotype replacement has also been observed for non-PCV13 serotypes such as 22F or 10A [2,3]. A similar serotype replacement has been notified in other European countries [4,5] in contrast with a low rise of non-PCV13 serotypes found in the United States including a low burden of serotype 8 [6]. Among possible variations that may influence this difference are different vaccine schedules for PCVs with a 3 + 1 schedule in the United States vs the 2 + 1 schedule in Spain and other European countries or even geographical differences [7]. The on-going pursuit for more effective vaccines has resulted in the development of higher valent vaccines like PCV15, PCV20 and PCV21 yet the real-life impact of these newer formulations remains to be fully understood.

The pneumococcal capsule is the main virulence factor of the bacterium allowing pneumococcus to avoid the phagocytosis process. Among the wide variety of capsular polysaccharides, serotypes 3, 8 and 37 are associated with IPD and express a highly mucoid-type capsule [8]. This is important from the pathogenesis perspective because serotypes 3 and 8 are very prevalent and frequently related with case-fatality rates. With the notable exception of serotype 37 [9], the locus responsible for capsule polysaccharide synthesis, known as the *cap* or *cps* locus, has been identified between the *dexB* and *aliA* genes [10–13]. Most serotypes synthesize their capsule polysaccharide through a polymerase-dependent (*wzy*) mechanism, except for serotypes 3 and 37. Moreover, capsule regulation is intricate, encompassing transformation and recombination events that facilitate capsule type-encoding gene exchange, a phenomenon known as capsular switching which holds significant implications as it can facilitate vaccine escape strains [14]. Additionally, certain pneumococcal serotypes exhibit spontaneous colony phase variants and the appearance of non-encapsulated mutants may play a role in the initial stages of pathogenesis, particularly in biofilm formation and attachment [15].

Therein, the surveillance and molecular characterization of highly prevalent non-PCV emerging serotypes, such as serotype 8, is crucial. Here, we describe the evolution of serotype 8 strains causing IPD in children and adults in Spain during the last 14 years (2009–2023), studying the main lineages (considering Sequencetype, ST; Clonal Complex, CC; and Global Pneumococcal Sequence Cluster, GPSC), the capsular cluster, and the pathogenesis of circulating clinical isolates.

## Materials and methods

### Study design

We characterized 34 357 IPD isolates from January 2009 to December 2023 (Supplemental Figure S1). Serotyping was performed using Quellung reaction, dot blot assay using specific antisera, and/or polymerase chain reaction (PCR)-capsular sequence typing as previously described [2]. In total, we notified 4377 IPD-serotype 8 cases from 2009 to 2023. The epidemiological evolution of serotype 8 was analysed for different age groups covering the paediatric and adult populations including whole genome sequencing (WGS) analysis and pathogenesis studies (Supplemental Figure S1).

### Whole genome sequencing and bioinformatic analysis

Chromosomal DNA was obtained using the genomic DNA purification kit Wizard (Promega). Illumina sequencing libraries were prepared using the Illumina DNA prep (96) and IDT for Illumina Nextera DNA unique dual indexes and then sequenced at the Genomic Unit at ISCIII using Novaseq 6000, which produced 2 × 150 bp paired-end read data. Reads were deposited at the European Nucleotide Archive (Supplemental Table S1).

WGS was performed using the following workflow (Supplemental Table S2). Read quality control (FASTQC), removal of low-quality sequences (Trimmomatic), elimination of possible contaminations from other bacterial species, and *de novo* assembly of read using SPAdes were performed using the pipeline INNUca (https://github.com/B-UMMI/INNUca). MLST profile was determined using the mlst tool (https://github.com/tseemann/mlst), and global contextualization with lineage assignation through GPSC was performed using PathogenWatch (https://pathogen.watch/). For antimicrobial resistance and virulence factor profiling we used abricate (https://github.com/tseemann/abricate). PBP typing was also performed (https://github.com/rpetit3/pbptyper). Genomes and Pan-genome were analysed using Prokka (https://github.com/tseemann/prokka) and Roary respectively (https://sanger-pathogens.github.io/Roary/). roProfile was used to compare genes between different lineages (https://github.com/cimendes/roProfile). Single nucleotide polymorphism (SNP) analysis was performed using Snippy (https://github.com/tseemann/snippy), phylogenetic trees were constructed using RaxML-NG (https://github.com/amkozlov/raxml-ng), and genome-wide prediction of recombination was analysed using Gubbins (https://github.com/nickjcroucher/gubbins). Mutations in the capsular operon were characterized using Geneious R9 software (Biomatters) and strain 573/62-CR931644 as reference strain [12] We also used Geneious R9 to identify mutations and acquisition of resistance genes and to closely analyse the main virulence factors of *S. pneumoniae*, using references annotated in TIGR4, D39, R6, and OXC141 genomes. Virulence factors were grouped into alleles for comparison purposes. Prophages were classified using the pneumococcal prophages (PPH) families previously described [16]. We used ICEberg 3.0 to search for genetic mobility elements (https://tool2-mml.sjtu.edu.cn/ICEberg3/). We classified all isolates in ST, CC, and lineage (GPSC).

### Interaction of *S. pneumoniae* with human epithelial cells

We followed previously described methods for adhesion assays using the A549 cell line [17]. Briefly, 10^5^ cells were infected with 2 × 10^6^ CFU of pneumococcal cells [multiplicity of infection (MOI) of 1 A549:25 S. *pneumoniae*)] and incubated at 37°C in a 5% CO_2_ atmosphere for 1 h. Then, infected cells were washed three times and 0.025% PBS-saponin was added to gently gently lyse the cells. Results are based on viable bacteria counts recovered in blood agar plates.

### Biofilm formation assays

Biofilm formation was analysed using treated 96-well polystyrene microtiter plates as previously described [18]. Cells were cultured in C + Y medium adding per well a concentration of 4.5 × 10^6^ CFU/mL. For crystal violet (CV) staining, after a 5-h incubation period, the total growth (*A*_595_) was measured using the BioTek Epoch 2 reader. The biofilm biomass was solubilized with 95% ethanol and quantified by measuring the *A*_595_ using the same reader.

### Recruitment of factor H assays

We followed previously described methodology [19]. Briefly, approximately 2 × 10^6^ CFU of strains of serotype 8 were incubated with 10 µL of HBSS as negative control or with 10 µL of serum diluted 1/6 from healthy individuals [All participants provided written informed consent (authorization approval of Ethics Committee: HULP: PI-1832). The project was approved by ISCIII Ethics Committee (Ref: CEI PI 45_2021-v2)]. After 20 min at 37°C, bacteria were washed twice with PBS-0.1% Tween 20 to remove unbound factor H, followed by incubation for 30 min on ice with sheep-anti-human factor H antibody (Serotec) diluted 1/300 in PBS. After two washes with PBS-0.1% Tween 20, bacteria were incubated for 30 min on ice with FITC-conjugated donkey anti-sheep antibody (Serotec) diluted 1/300 in PBS. Bacteria were then fixed in 3% paraformaldehyde (PFA) and analysed on a FACSCalibur flow cytometer. These results were expressed as a relative fluorescence index (RFI), which measures both the proportion of bacteria positive for factor H protein and the intensity of fluorescence that quantifies the bound immune component [19].

### Opsonophagocytosis assays (OPA)

We followed previously described methods for opsonophagocytosis assays (OPA) of *S. pneumoniae* using the HL-60 cell line (CCL-240; ATCC) differentiated into neutrophils [20]. For OPA we used 10^5^ HL-60 cells and 2.5 × 10^2^ CFU of *S. pneumoniae* strains (MOI of 400 HL-60:1 *S. pneumoniae*) that were previously opsonized for 1 h with 1/8 of baby rabbit serum a source of complement. Results are based on viable bacteria counts recovered in blood agar plates.

### Mice model of pneumococcal pneumonia

All experiments involving mice were conducted at the Instituto de Salud Carlos III (ISCIII) in compliance with Spanish legislation (RD 53/2013, ECC/566/2015) and the European Directive 2010/63/EU. Female BALB/c mice, 12 weeks old and approximately 20 grams in weight, were obtained from Charles River Laboratories. All procedures were carried out in accordance with the ethical standards set by the Bioethics and Animal Welfare Committee of ISCIII and the corresponding Regional Authorities, which approved the study protocols (PROEX 063.1/21). We followed previously described methodology for the mice pneumonia model [17]. Briefly, under anaesthesia with isoflurane, we infected groups of mice with serotype 8 clinical isolates from different lineages SPRLISCIII2746-11 (CC53/GPSC3), SPRLISCIII5174-16 (CC404/GPSC98), and SPRLISCIII1088-08 (CC63/GPSC9). Mice were intranasally challenged with 50 μL of bacterial suspension at a concentration of 5 × 10^5^ CFU per mouse. 24 h after infection, mice were sacrificed by CO_2_, and bacterial counts were determined from samples recovered from the lung. The results were expressed as CFU per millilitre of bacteria recovered from the lung.

### Statistical analysis

The corrected incidences were calculated as the number of IPD episodes per 100,000 population and year using population data from the Spanish National Statistical Institute as the denominator and the population capture of 80% to the denominator which is the typing coverage of our laboratory [2]. We assumed the same epidemiological characteristics for the population suffering from IPD not covered by our laboratory and no difference in the age distribution between the population covered by hospitals and the general population. We analysed the evolution of serotype 8 after PCV13 introduction in the private market (2010), or in the national paediatric immunization schedule (2016) and we also considered the impact of the COVID-19 pandemic (2020–2021), and the re-opening (2022–2023). Comparison of different periods was analysed by calculating the IRRs using Poisson regression models. Statistical analyses were performed using STATA v.14. For experimental procedures, data were collected from independent experiments, each with a minimum of three replicates. To compare two groups, a two-tailed Student's *t*-test was employed, while one-way ANOVA with Dunnett's post hoc test was used for multiple comparisons. All analyses were performed using GraphPad InStat version 8.0. Significance was defined as *P* < 0.05 (*), with *P* < 0.01 (**) and *P* < 0.001 (***).

## Results

### Increase of serotype 8 in children and adults appeared after the paediatric use of PCV13

**Figure 1.**
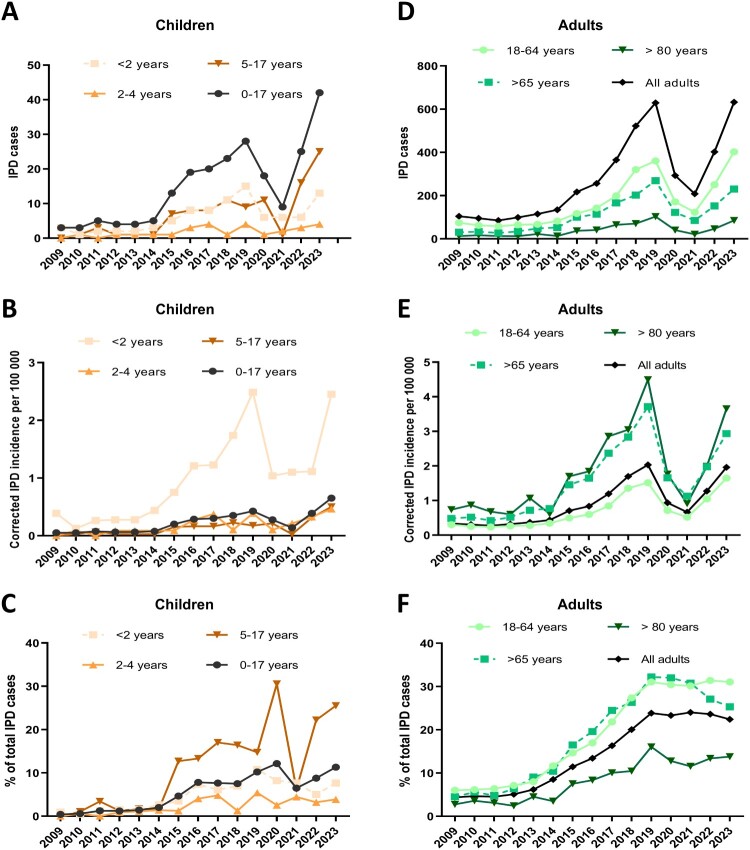
Trends of IPD by serotype 8 in Spain for different age groups during 2009–2023. Paediatric population (A–C). Adult population (D–F). Number of IPD cases (A, D), corrected IPD incidence per 100,000 habitants (B, E) and percentage of IPD cases by serotype 8 in relation total paediatric or adult IPD (C, F).

In Spain, PCV13 was implemented in 2016 in the national immunization paediatric calendar (2 + 1 schedule) with high vaccination coverages in children (> 95%) even during the COVID-19 pandemic, although from 2010 to 2015 it was used in the private market with good coverage rates (67–82%) [2]. In immunocompetent adults aged ≥ 65 years old, the use of PPV23 that include serotype 8, was the general recommendation by the Spanish Ministry of Health since 2004 although several regions started using PCV13 for adults since 2016 [2]. Our results show that during the early period after PCV13 private use the burden of IPD by serotype 8 remained very low in children and adults (Figure 1). However, coinciding with the generic use of PCV13 in children in 2015/2016, the incidence of IPD by serotype 8 increased markedly in the paediatric and adult populations reaching its maximum peak in 2019. In children, the increase of serotype 8 was higher in the age grou*p* < 2 years old (IRR, 8.27; 95% CI, 3.24–21.15) when comparing 2017–2019 vs. 2010–2012 (Figure 1A-B and Table 1). In adults, the rise of serotype 8 affected all age groups (104 cases in 2009 vs. 629 in 2019 for all ages or 30 cases in 2009 vs. 269 in ≥65 years old) despite the recommended use of the PPV23 for adults over 65 years old, confirming the lack of protection of this vaccine against this serotype (IRR, 6.18; 95% CI, 4.29–8.92) when comparing 2017–2019 vs. 2009 (Figure 1D-F and Table 1).

**Table 1.** Number of cases, corrected incidence, and incidence rate ratios (IRRs) of IPD by serotype 8 in 2022–2023 compared with 2017–2019 and 2020–2021; and 2017–2019 compared with 2009, 2010–2012, 2013–2016, and 2020-2021.

	2009		2010–2012		2013–2016		IRR 2017–19 vs. 2009	95% CI	IRR 2017–19 vs. 2010–12	95% CI	IRR 2017–19 vs. 2013–16	95% CI
	Pre-PCV13		Early vaccine effect		Middle vaccine effect							
	Cases	Corrected incidence (per 100 000)	Cases	Corrected incidence (per 100 000)	Cases	Corrected incidence (per 100 000)						
< 2 years	3	0.38	5	0.22	18	0.66	4.70	1.44–15.3	8.27	3.24–21.15	2.74	1.55-4.85
2–4 years	1	0.09	2	0.06	6	0.13	3.35	0.42–26.43	5.22	1.13–24.16	2.19	0.78-6.16
5–17 years	1	0.02	5	0.04	17	0.09	8.66	1.18–63.66	5.29	2.04–13.7	2.14	1.17-3.91
0–17	3	0.05	12	0.06	41	0.15	7.79	2.45–24.73	5.94	3.22–10.95	2.32	1.58-3.4
All Adults	104	0.34	279	0.30	722	0.59	4.87	3.99–5.94	5.48	4.83–6.23	2.80	2.56-3.06
18–64 years	74	0.30	185	0.25	408	0.43	4.12	3.25–5.22	4.94	4.22–5.79	2.91	2.59-3.27
≥ 65 years	30	0.48	94	0.48	314	1.15	6.18	4.29–8.92	6.15	4.95–7.63	2.58	2.25-2.95
≥ 80 years	13	0.73	41	0.71	113	1.32	4.72	2.7–8.26	4.87	3.5–6.78	2.63	2.11-3.3
Total IPD by serotype 8	107	0.29	291	0.26	763	0.51	4.94	4.06–6.01	5.50	4.85–6.23	2.77	2.54-3.02
	2017–2019		2020–2021		2022–2023		IRR 2020–21 vs. 2017–19	95% CI	IRR 2022–23 vs. 2020–21	95% CI	IRR 2022–23 vs. 2017–19	95% CI
	Late vaccine effect		COVID-19 pandemic		Reopening							
	cases	Corrected incidence (per 100 000)	cases	Corrected incidence (per 100 000)	cases	Corrected incidence (per 100 000)						
< 2 years	34	1.80	12	1.07	19	1.78	0.59	0.31–1.14	1.66	0.81–3.42	0.98	0.56-1.73
2–4 years	9	0.29	3	0.15	7	0.39	0.53	0.14–1.95	2.56	0.66–9.9	1.35	0.5-3.63
5–17 years	28	0.19	12	0.12	41	0.41	0.64	0.32–1.25	3.41	1.79–6.5	2.17	1.34-3.51
0–17	71	0.36	27	0.21	67	0.52	0.58	0.37–0.9	2.52	1.61–3.95	1.45	1.04-2.03
All Adults	1516	1.64	501	0.80	1034	1.62	0.49	0.44–0.54	2.03	1.83–2.26	0.99	0.91-1.07
18–64 years	879	1.24	294	0.62	652	1.35	0.50	0.44–0.57	2.19	1.91–2.52	1.09	0.98-1.21
≥ 65 years	637	2.98	207	1.39	382	2.48	0.46	0.4–0.54	1.79	1.51–2.12	0.83	0.73-0.95
≥ 80 years	238	3.47	61	0.81	131	2.83	0.23	0.18–0.31	3.48	2.57–4.72	0.81	0.66-1.01
Total IPD by serotype 8	1587	1.41	528	0.70	1101	1.43	0.49	0.45–0.54	2.06	1.86–2.29	1.01	0.94-1.1

During the COVID-19 pandemic (2020–2021), the burden of IPD caused by serotype 8 rapidly decreased up to levels found in the early PCV13 period (Figure 1) except in children < 2 years old with similar rates to 2015/2016 (Figures 1(A,B)). This decrease in IPD incidence in all populations was statistically significative. In the re-opening period, we observed a complete resurgence and exceeded levels of serotype 8 IPD in the paediatric population (2022–2023 vs. 2017–2019 IRR, 1.45; 95% CI, 1.04–2.03), with a significant increment in the population aged 5–17 compared to pre-pandemic levels (2022–2023 vs. 2017–2019 IRR, 2.17; 95% CI, 1.34–3.51; Figure 1(B) and Table 1). In adults, we found a partial resurgence of cases in 2022, and a complete revival in 2023, showing a similar situation to the pre-pandemic period (2022-2023 vs. 2017–2019 IRR, 0.99; 95% CI, 0.91–1.07; Figure 1(E,F) and Table 1).

### Diversity of serotype 8 population including pangenome characterization and associated virulence factors

**Figure 2.**
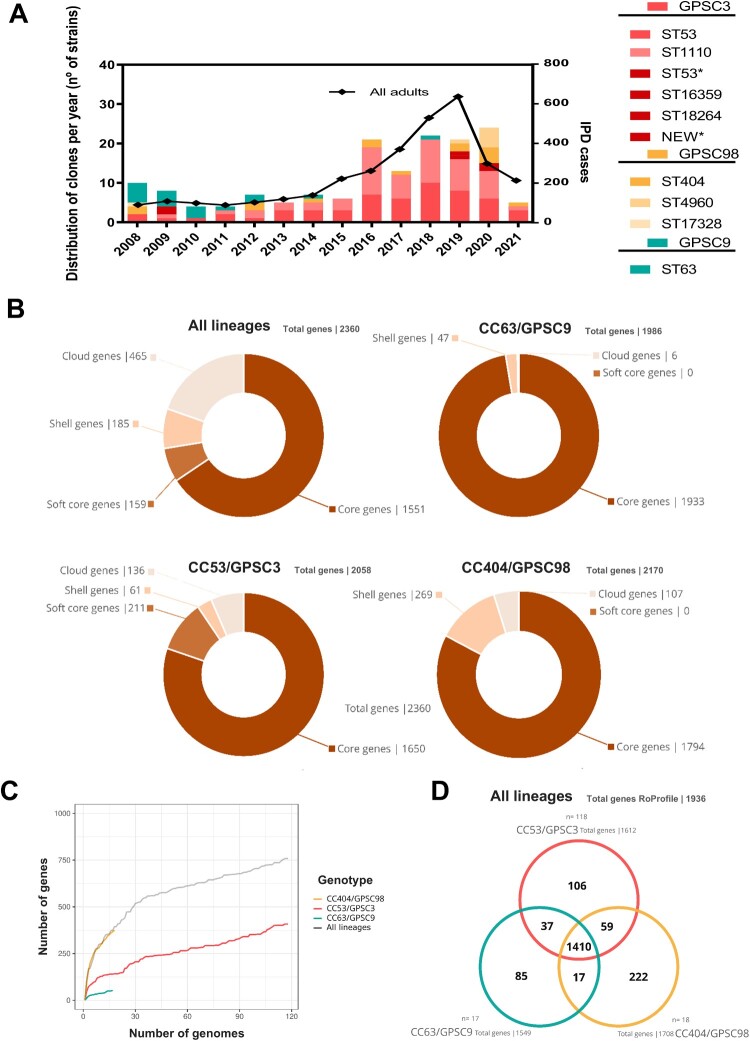
Molecular characterization of serotype 8 in Spain. Distribution of serotype 8 clones (STs) per year grouped by lineage (GPSC) (A). Pangenome showing the percentage of core and accessory genome of each serotype 8 lineage (B). Relationship analysis between the total and conserved genes. The X-axis shows the number of genomes and the Y-axis the difference between the total and conserved genes in the pangenome analysis for each added genome (C). Presence/absence genes analysis by lineage using roProfile where CC53/GPSC3, CC404/GPSC98, and CC63/GPSC9 are represented in red, yellow, or blue respectively (D).

Molecular characterization by WGS unveiled the existence of three circulating lineages within serotype 8 in Spain: CC53/GPSC3 as the predominant lineage (77.1%) and two minority lineages; CC404/GPSC98 (11.8%), and CC63/GPSC9 (11.1%) (Figure 2). Most CC63/GPSC9 isolates belonged to the pre-PCV13 and early PCV13 periods (2008-2012) (Figure 2) with a notorious presence due to the spread of multidrug-resistant serotype 8 strains, affecting up to seven Spanish regions with high relevance in Madrid [21]. This was followed by a decrease of cases that explains the reduction in the MDR phenotype among serotype 8 (Supplemental Figure S2). Since 2011, CC53/GPSC3 has been the predominant lineage in Spain, with a similar distribution between ST53 and ST1110 (Figure 2).

The overall serotype 8 pangenome analysis classified up to 72% of the genes as soft core and core genes (core genome) (Figure 2(B)). The remaining 28% were shell and cloud genes (accessory genome). When analysing individual lineages, over 90% of CC53/GPSC3 and 97% of CC63/GPSC9 pangenomes were soft core and core genes. In contrast, only 82.67% of CC404/GPSC98 genes were soft core and core genes, showing the highest variability within serotype 8. The relationship between the total and conserved genes indicates that CC53/GPSC3 and CC63/GPSC9 lineages had more conserved genomes, whereas CC404/GPSC98 has a more open genome (Figure 2(C)). Analysis evaluating the presence/absence of genes between lineages, confirmed that CC63/GPSC9 lineage shares 93.42% of genes content with the CC53/GPSC3, whereas CC404/GPSC98 shares only 86% of its genes with the CC53/GPSC3 (Figure 2(D)).

**Figure 3.**
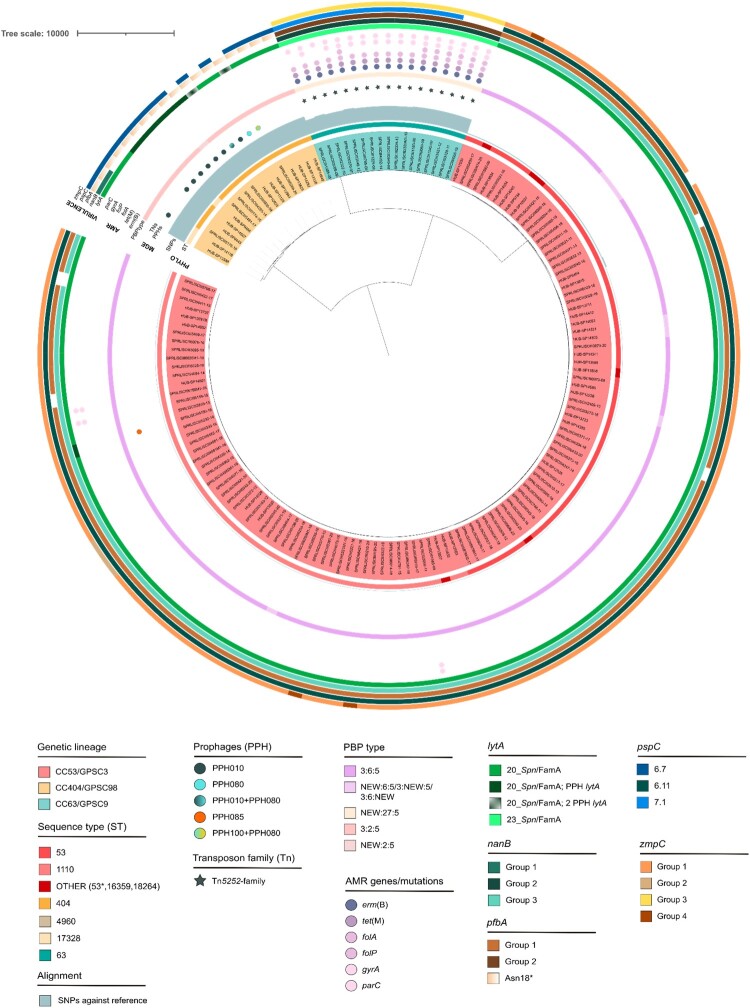
Phylogenetic tree of serotype 8 in Spain. The phylogenetic tree was obtained using the snippy, snippy-core, and Gubbins steps described in methods, and groups genomes into different clades based on the SNPs against the reference (oldest CC53/GPSC3 genome). Annotated data about the genetic lineage, vaccine period, number of SNPs against reference, PBP type, antimicrobial resistance genes/mutations found, and virulence factors can be observed in the phylogenetic tree and are detailed in the legend. PHYLO, phylogeny data; MGE, mobile genetic elements; AMR, antimicrobial resistance; VIRULENCE, virulence factors.

When all the genomes were compared with the oldest CC53/GPSC3 genome in our study (SPRLISCIII0973-08), only minor variability within GPSC3 lineage was found, and more distant genetic relationship between GPSC98 and GPSC3 than between GPSC9 and GPSC3 (Figure 3).

Analysis of antibiotic susceptibility revealed that the majority of serotype 8 isolates within CC53/GPSC3 and CC404/GPSC98 were susceptible, except for three isolates harbouring *gyrA* and *parC* mutations, conferring resistance to quinolones. However, all CC63/GPSC9 isolates exhibited resistance determinants, leading to macrolides resistance [*erm*(B)], tetracycline [*tet*(M)], trimethoprim (*folA*), and quinolones (*parC*). Some strains also presented *folP* and *gyrA* mutations (Figure 3 and Supplemental Table S1). In all CC63/GPSC9 isolates, *erm*(B) and *tet*(M) genes were carried in mobile genetic elements of the Tn*5252*-family. Interestingly, none of the isolates had mutations in PBPs that could be associated with β-lactam resistance (Supplemental Table S1).

Moreover, we found differences between lineages in the presence/absence of prophages and in important virulence factors (Figure 3 and Supplemental Table S3). We found that CC404/GPSC98 isolates did not harbour the *zmpC* (IgA1 and matrix metalloprotease) and *nanB* genes (neuraminidase) and presented a truncated PfbA protein (plasmin – and fibronectin-binding protein) compared to the other two main lineages (Figure 3 and Supplemental Table S3). Three different PspC alleles in serotype 8 isolates, each one associated with a different GPSC, were detected. All CC53/GPSC3 isolates harboured *pspC* 6.11, most CC404/GPSC98 isolates harboured *pspC* 6.7, and most CC63/GPSC9 isolates harboured *pspC* 7.1 (Figure 3 and Supplemental Table S3). However, *pspC* could not be detected in six isolates belonging to either CC404/GPSC98 or CC63/GPSC9. Finally, we found that all serotype 8 isolates have family A *lytA* alleles, and most CC404/GPSC98 isolates also presented a PPH_*lytA* (Figure 3).

### Non-mucoid colony variants in serotype 8 strains causing IPD

**Figure 4.**
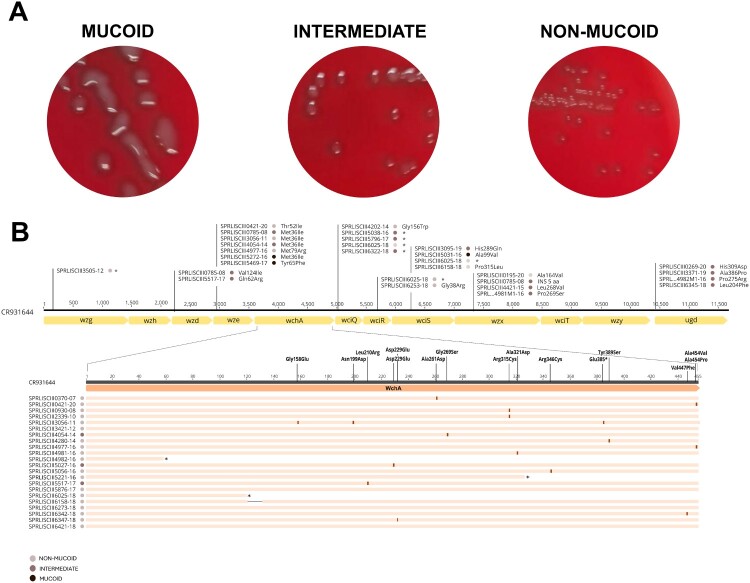
Relationship between variations in the mucoid morphology of serotype 8 strains and mutations in the capsular operon. Colony morphology of mucoid, intermediate, and non-mucoid isolates of serotype 8 (A). Representation of strains showing non-synonymous SNPs, indels, and missense SNPs producing changes in the proteins of the capsular operon of serotype 8 using the strain 573/62 (CR931644) as a reference [12]. Black, brown and light brown circles represent mucoid, intermediate and non-mucoid phenotypes, respectively. Asterisk indicates the truncation of the protein (B).

As mentioned above, the mucoid phenotype of the colonies on blood agar plates is characteristic of serotypes 3, 8 and 37 clinical isolates. However, we observed that serotype 8 clinical isolates can also display an intermediate or non-mucoid phenotype (Figure 4(A)), even within the same sample (mixed colonies). These intermediate and non-mucoid serotype 8 isolates are still typed via Quellung reaction, dot blot assay using specific antisera, and PCR-capsular sequence typing. In the last two full epidemiological years presented in the manuscript (2022–2023), we received approximately 64.9–67.7% mucoid, 8.9–14.7% intermediate, 17.6–21.1% non-mucoid, and 2.3–2.8% mixed-colony serotype 8 isolates (colonies with mucoid, intermediate, and non-mucoid morphologies on the same plate).

The in-depth study of the capsular phenotype of 111 isolates by visual observation classified them as mucoid (68 isolates), intermediate (20 isolates), and non-mucoid (23 isolates). Non-synonymous single nucleotide polymorphisms (SNPs), indels, and missense SNPs found in the capsular operon of serotype 8 genomes are depicted as amino acid changes (Figure 4(B) and Supplemental Table S4). SNPs that produced synonym mutations were discarded in the analysis. Furthermore, no SNPs were found in the core promoter (neither in the −10 nor −35 boxes), except for variability in the number of adenines in the spacing sequence behind the promoter (position −63 relative to the first codon of the *wzg* gene), unrelated to the phenotype; neither in the sequences 37_CE and 31_CE implicated in the regulation of the biosynthesis of the capsular operon. Additionally, a nucleotide change (G→A) at position −169 in the spacing sequence was observed only in two clinical isolates. Moreover, we observed that most non-mucoid clinical isolates (19 out of 23) and those with intermediate phenotype (14 out of 20) have non-synonymous SNPs or SNPs and indels leading to truncation of capsular operon genes (*wzg*, *wchA*, *wciQ*, *wciR* and *wciS*) (Figure 4(B) and Supplemental Table S4). Specifically, most non-mucoid isolates had mutations in the *wchA* gene (previously named *cap8E*) [11], which encodes the initial transferase essential for capsular polysaccharide synthesis [11, 12], In contrast, mucoid clinical isolates barely presented any non-synonymous SNPs, with only three mucoid isolates exhibiting them only in *wze* and *wciS* genes (3 out of 68 mucoid isolates) (Supplemental Table S4).

### CC53/GPSC3 is associated with increased evasion of phagocytosis and disease potential

**Figure 5.**
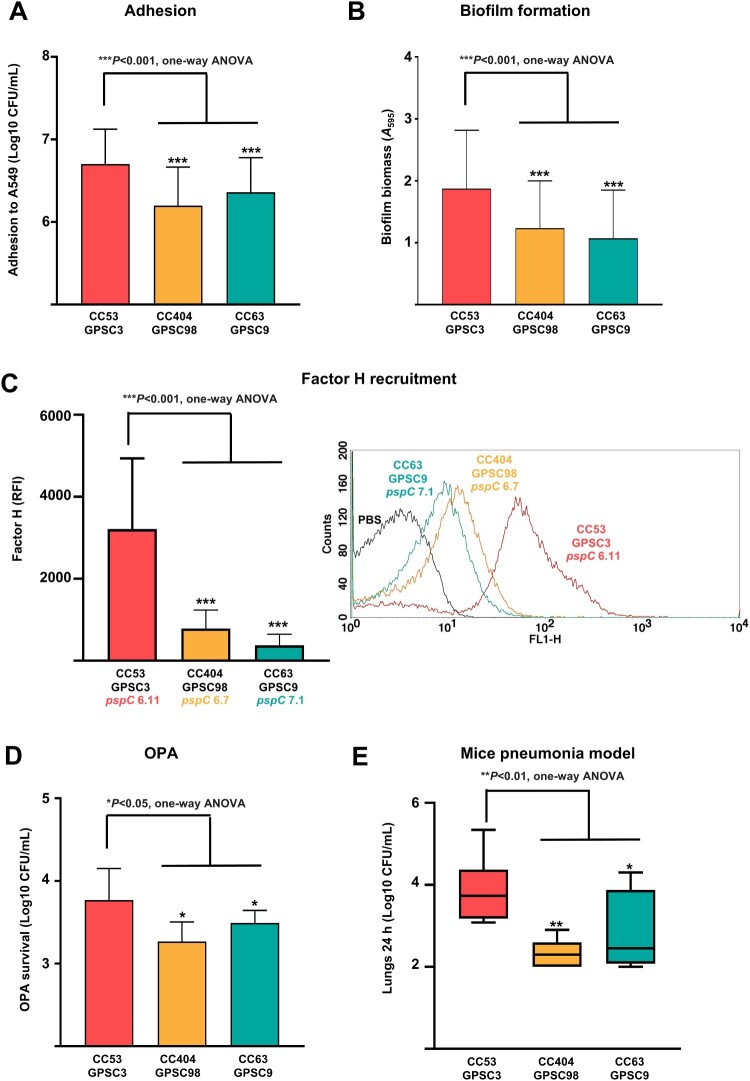
Differences in pathogenesis between circulating clones of serotype 8. Adhesion to human lung epithelial cells (A549 model) (A). Biofilm formation (B). Recruitment of the complement downregulator factor H measured by flow cytometry. Data expressed as relative fluorescence index (RFI). Example of a flow cytometry histogram (C). Evasion of opsonophagocytosis (OPA) (D). Mice pneumonia model. Viable colony counts expressed as Log_10_ CFU/ml from the lung of mice infected with serotype 8 strains from different lineages (E). Error bars represent standard deviations, and asterisks mark statistically significant results (two-tailed Student’s *t*-test: *P < 0.05; **P < 0.01; ***P < 0.001). For multiple comparisons, one-way ANOVA was performed.

We studied different aspects of the host–pathogen interaction such as adhesion to human lungs, biofilm formation, recruitment of the complement fluid-phase downregulator factor H, phagocytosis rates, and a mice pneumonia model to explain the emergence of CC53/GPSC3 as the dominant lineage in the last years and the potential relevance of mucoid vs. non-mucoid strains within serotype 8 (Supplemental Figure S1 and Figure 5). Adhesion to human lung epithelial cells and biofilm formation confirmed that isolates of CC53/GPSC3 had an increased ability to adhere to the lung epithelium and form biofilms compared to other lineages (Figure 5(A,B)). When analysing the interaction with the immune system, we observed that CC53/GPSC3, which had a distinct *pspC* allele (6.11), recruited an increased proportion of factor H compared to the other lineages (Figure 5(C)). Moreover, CC53/GPSC3 evaded the phagocytosis better than the other lineages showing that, in serotype 8, the genetic background contributes to evasion of the immune system (Figure 5(D)). Finally, to evaluate the *in vivo* relevance of our findings in an experimental mice pneumonia model, we used three strains from the different genetic background (CC53/GPSC3, CC404/GPSC98 and CC63/GPSC9). Bacterial counts recovered from the lung at 24 h showed that mice infected with the CC53/GPSC3 strain presented a higher bacterial load in the lungs (Figure 5(E)), confirming the increased virulence of this lineage compared to CC404/GPSC98 and CC63/GPSC3.

**Figure 6.**
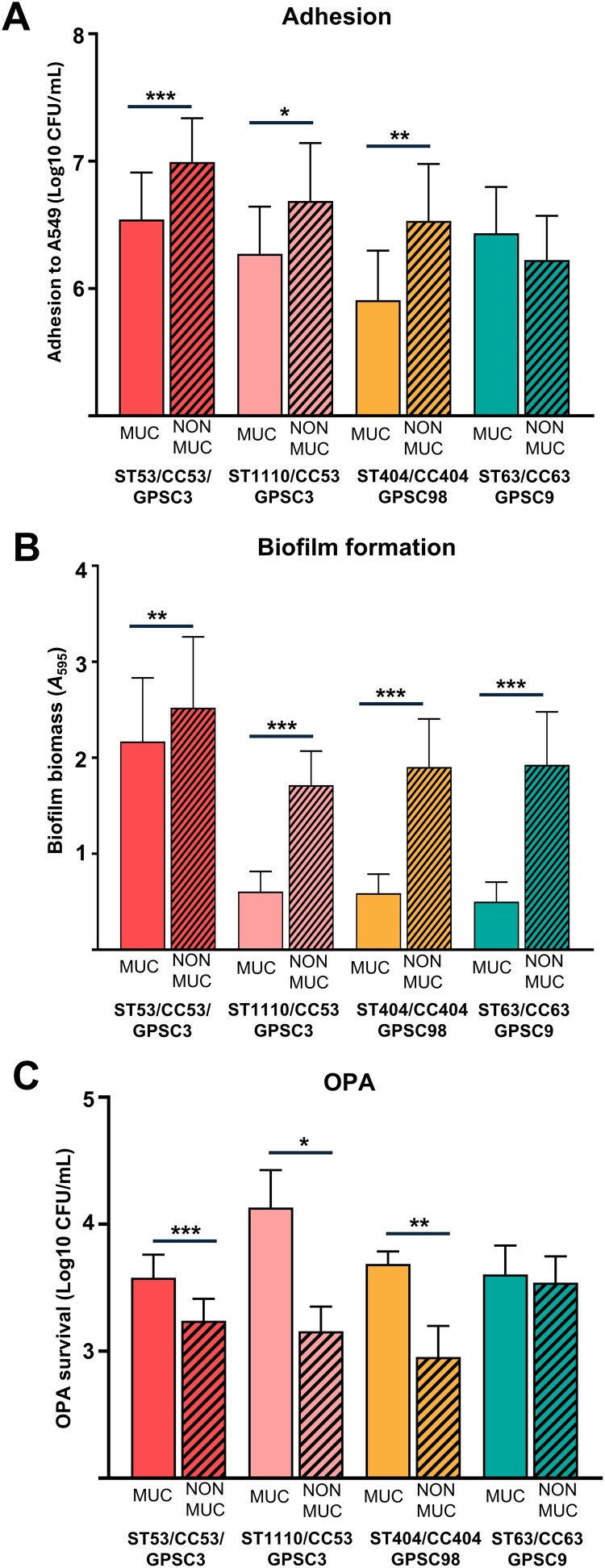
Differences in pathogenesis between phenotypes of serotype 8. Comparison between mucoid phenotypes within ST-lineages. Adhesion to human lung epithelial cells (A549 model) (A). Biofilm formation (B). Evasion of opsonophagocytosis (OPA) (C). Error bars represent standard deviations, and asterisks mark statistically significant results (two-tailed Student’s *t*-test: *P < 0.05; **P < 0.01; ***P < 0.001). For multiple comparisons, one-way ANOVA was performed.

Experiments comparing mucoid vs. non-mucoid strains of the different lineages confirmed that the mucoid phenotype exhibits a greater resistance to phagocytosis (Figure 6(C)). This effect was consistent in most lineages-STs within serotype 8. Colonization studies including interaction with human lung cells and biofilm formation indicated that non-mucoid variants had a higher ability to adhere the lung and form biofilms (Figure 6(A,B)).

## Discussion

Serotype replacement by non-vaccine serotypes is a frequent phenomenon that occurs after PCV introduction in countries with high coverage rates [2]. In this study, we have evaluated the impact of PCV13 and the COVID-19 pandemic in the epidemiology of serotype 8 pneumococci showing a promptly increase after the private use of PCV13, and a marked rise after the implementation of this vaccine in the paediatric vaccination calendar. These results align with recent reports from other European countries, which highlight the relevance of this serotype as one of the most prevalent following the pandemic and establish a starting point to assess the effectiveness of the new PCV20 and PCV21 vaccines against this serotype in the coming years [2]. During the COVID-19 pandemic, mainly due to the implementation of non-pharmacological interventions (NPIs) such as facial masks, social distancing and lockdowns, we observed a drastic decrease in IPD incidence by serotype 8 in all populations. The decrease was not unique to this serotype, as we observed a total reduction of IPD by all serotypes in Spain during the COVID-19 pandemic [2]. The decrease of IPD and other invasive bacterial diseases due to NPIs during 2020–2021 was observed worldwide [22], but after the reopening (2022-2023) we observed an upsurge of IPD cases by serotype 8 (this study) and other serotypes [2]. The marked upsurge of this serotype in children and adults in 2023 with IPD burden comparable to the last pre-pandemic year suggests that the use of vaccines such as PCV10, PCV13 or PCV15 that do not include this serotype in their formulations may not be sufficiently protective and that broader vaccines are needed [2]. As previously analysed in other epidemiological studies evaluating national data of IPD [2,3], we did not adjust IPD incidence and cases considering cofounding factors such as comorbidities or regional vaccine coverage. The increase in serotype 8 occurred in a context of varying vaccine coverage depending on the target population. While vaccination coverage with PCV13 was high among children, coverage with PCV13 or PPV23 remained low among Spanish adults [2]. Herd protection was consistent during the study period, as PCV13 was used with similar coverage rates across different regions. Generally, all regions using either PCV13 or PPV23 for adults had comparable urban/rural population mixes and similar age distributions. In this regard, a previous study by our group [3] showed that regions administering PCV13 to adults experienced a greater reduction in IPD cases caused by PCV13 serotypes compared to those using PPV23. Regarding comorbidities, chronic conditions were associated with an increased risk of hospitalization among adults in Spain, particularly in the 18–64 age group [23].

Molecular characterization of adult isolates demonstrated the presence of three main lineages within serotype 8 in the last 15 years. The lineage ST63/CC63/GPSC9 associated with multidrug resistance was derived from a capsular switching event from the Sweden^15A^-ST63 PMEN clone [21]. However, the expansion of this GPSC9 lineage was limited probably due to the cost that antibiotic resistance imposes to bacterial fitness as it has been previously reported in *S. pneumoniae* [24]. After 2011, IPD incidence by serotype 8 rapidly increased with CC53/GPSC3 becoming the main circulating lineage in Spain, in line with previous reports [25].

In the case of CC404/GPSC98, molecular analyses confirmed loss of relevant virulence factors involved in adhesion and invasive disease such as ZmpC, NanB and PfbA, in comparison to GPSC3. This finding could explain its lower prevalence. Defective strains in ZmpC have been associated with lower potential to establish pneumonia, mutants in NanB have impaired adhesion to human epithelial cells including a reduced ability to successfully colonize the respiratory tract, and mutants in PfbA are associated with lower adhesion and antiphagocytic activity [26–28]. Moreover, pneumococci of the CC404/GPSC98 lineage had a more open pangenome and contained PPHs, in contrast to the other lineages. All these characteristics could explain why CC404/GPSC98 was less prevalent and might be associated with a more colonization/non-invasive disease niche [25].

Our results confirmed that, since PCV13 introduction in 2010, the dominant serotype 8 lineage circulating in Spain is CC53/GPSC3. Genomic analysis demonstrated that this lineage does not contain antibiotic resistance factors (as GPSC9 does) or lacks important virulence factors as GPSC98. Another differential aspect that could explain the predominance of this lineage in the last years is the expression of a different PspC allele. This is not surprising because the *pspC* locus is highly polymorphic [29]. All the clinical isolates of CC53/GPSC3 harboured *pspC* 6.11 whereas CC404/GPSC98 isolates and CC63/GPSC9 had *pspC* 6.7 or *pspC* 7.1 respectively and even some strains of these two minor lineages lacked a *pspC* gene. This may be of great relevance in virulence because PspC plays critical roles in several aspects of the pathogenesis process: (i) PspC recruits factor H and therefore avoids complement-mediated immunity [30]; (ii) it is involved in the binding to secretory IgA and the polymeric IgG receptor on epithelial cells [31], and (iii) it participates in biofilm formation by triggering the hyper adhesive property of the biofilm [32,33]. In addition, pneumococcal strains expressing different *pspC* alleles, display different phenotypes in the host–pathogen interplay showing different virulence patterns in mice [30,34,35]. To further assess this finding, we performed factor H recruitment assays observing that CC53/GPSC3 harbouring *pspC* 6.11 presented an enhanced recruitment of factor H than the other lineages. Overall, our results suggest that the increased adhesion to human lung cells, biofilm formation, factor H recruitment, opsonophagocytic evasion, and mice virulence in the predominant lineage CC53/GPSC3 might be related to the expression of a different *pspC* allele in comparison to the less prevalent lineages. Hence, the CC63/GPSC9 lineage that promptly disappeared after PCV13 introduction displays *pspC* 7.1 that is not involved in IPD as several pneumococcal strains lacking this allele of *pspC* were not attenuated in virulence [35]. The higher potential of CC53/GPSC3 isolates to cause disease in our study agrees with a recent report showing increased hypervirulence of a serotype 8 strain of ST53 producing meningitis in rats [36]. A potential limitation of the present study is that pathogenesis experiments were conducted using a limited number of strains, as it is not feasible to perform these experiments with a larger number of strains. However, our experiments contained at least two strains from each lineage and the most prevalent STs and up to three different replicates were performed.

Another important finding of the current study is the discovery of mucoid and non-mucoid phenotypes within serotype 8 strains in all the lineages characterized. We found that the majority of serotype 8 isolates with a non-mucoid/intermediate phenotype presented non-synonym SNPs or SNPs and indels leading to truncation of capsular operon proteins. Specifically, these mutations were concentrated in the *wchA* gene which is consistent with previous data showing that naturally occurring *wchA* mutations were responsible for the non-typable phenotype in pneumococci [37] and in non-invasive isolates with high levels of biofilm formation from ocular infections [38]. Our results showing that non-mucoid strains had increased susceptibility to phagocytosis, higher adhesion and better biofilm-forming capacity are compatible with a less invasive and higher colonizing phenotype. Moreover, point mutations in *cap8E/wchA* have been described in serotype 8 after consecutive culture passes, being associated with a reversible phase variation phenomenon [39], which agree with the observation that in some strains of serotype 8 mucoid and non-mucoid colonies of the same clinical isolate can be found on blood agar plates. One reasonably explanation is that the versatility of pneumococcal strains of serotype 8 expressing different capsular levels is an evolutionary advantage for the pathogenesis process displaying the non-mucoid phenotype in colonization and early phases of attachment and the mucoid phenotype during the invasive disease stage [15]. During carriage or attachment to the lower respiratory tract, non-mucoid phenotype may be more important whereas during the systemic dissemination, the bacterium prevents the host immune response by increasing the capsule production [40]. It is important to consider that an equilibrium between capsule amount and pathogenesis can occur depending on the pathogenesis stage and although non-mucoid strains may be less virulent, they express capsule but in a lower quantity and are still virulent [15]. One limitation regarding the underlying mechanisms is the unclear relevance of the different mutations in the capsular operon (including *wchA*) in terms of pathogenesis. Future experiments using capsule mutants with the described mutations, as well as *pspC* mutants with different alleles, would be useful to more precisely evaluate their contribution to virulence.

Overall, our study confirms the relevance of serotype 8 as one of the most prevalent serotypes causing IPD in Spain with a marked increase after the use of PCV13. Although three lineages were identified, the CC53/GPSC3 was the dominant lineage that spread after PCV13 introduction with a high potential to produce respiratory infections. The ability to evade phagocytosis, recruitment of factor H, adhesion to human cells and biofilm formation could help this lineage to increase its invasiveness and became an important cause of respiratory infections. The higher virulence capacity of CC53/GPSC3 strains that have emerged after PCV13, might explain the rise of serotype 8. Hence, countries without reporting serotype replacement by serotype 8 might be due to the predominance of lineages with lower disease potential. Diverse phenotypic variants within serotype 8 may confer an advantage in causing different pathologies. The use of PCVs of broader spectrum including serotype 8 would be very useful to control the rise of this serotype in children and adults.

## Supplementary Material

SUPPLEMENTALMAT_DEF.pdf

## Data Availability

All reads used for WGS analysis were deposited at the European Nucleotide Archive project (Accesion PRJEB81522, Supplemental Table S1). All epidemiological and experimental data requests should be submitted to JS (jsempere@isciii.es) or JY (jyuste@isciii.es). Requests will be assessed for scientific rigour before being granted, and a data-sharing agreement might be required.
