# Supplementary material for: The rise of serotype 8 is associated with lineages and mutations in the capsular operon with different potential to produce invasive pneumococcal disease
Source: Emerg Microbes Infect. 2025 Jun 16;14(1):2521845. doi: 10.1080/22221751.2025.2521845 (PMC12231264; doi:10.1080/22221751.2025.2521845)
Supplement: SUPPLEMENTALMAT_DEF.pdf [file TEMI_A_2521845_SM5165.pdf]

SUPPLEMENTAL MATERIAL

The rise of serotype 8 is associated with lineages and mutations in the capsular operon with different potential to produce invasive pneumococcal disease

Covadonga Pérez-García[a,1], Aída González-Díaz[b,c,1], Mirian Domenech[a,c], Mirella Llamosí[a], Aída Úbeda[a,c], Juan Carlos Sanz[d,e], Ernesto García[f], Carmen Ardanuy[b,c], Julio Sempere[a,c,]*, Jose Yuste[a,c,]*.

Content

Figure S1: Study design and strains used in the different studies. **2**

Table S1: Genomes analyzed in the study, accession numbers, metadata and main antibiotic resistance and virulence factors. **3-9**

Table S2: Bioinformatic workflow used for WGS analysis. **10**

Figure S2: Evolution of serotype 8 strains harboring multidrug resistance (MDR) to erythromycin, clindamycin and tetracycline during 2008-2021 **11**

Table S3: Main differences in virulence factors between serotype 8 lineages. **12**

Table S4: Mucoid phenotype study showing polymorphisms that produce changes in proteins of the capsular operon. **13-14**

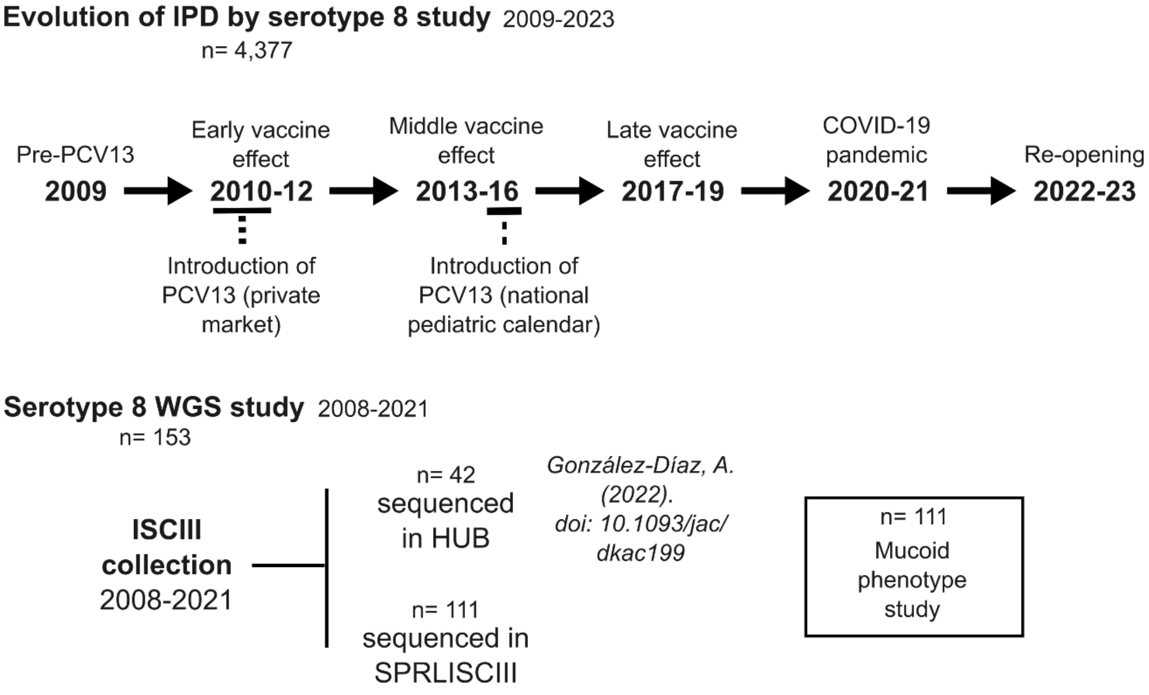

Serotype 8 pathogenesis study
n= 14

Isolate	Data	Genotype	Capsular phenotype	Reference
SPRLISCIII2409-10	Blood; adult	GPSC3/CC53/ST53	MUCOID	SPRL
SPRLISCIII2746-11	Blood; adult	GPSC3/CC53/ST53	MUCOID	SPRL
SPRLISCIII3505-12	Blood; adult	GPSC3/CC53/ST53	NON-MUCOID	SPRL
SPRLISCIII6273-18	Blood; adult	GPSC3/CC53/ST53	NON-MUCOID	SPRL
SPRLISCIII1383-09	Blood; adult	GPSC3/CC53/ST1110	MUCOID	SPRL
SPRLISCIII4981-16	Blood; adult	GPSC3/CC53/ST1110	NON-MUCOID	SPRL
SPRLISCIII1088-08	Blood; adult	GPSC9/CC63/ST63	MUCOID	SPRL
SPRLISCIII3421-12	Blood; adult	GPSC9/CC63/ST63	MUCOID	SPRL
SPRLISCIII0370-07	Blood; adult	GPSC9/CC63/ST63	NON-MUCOID	SPRL
SPRLISCIII2339-10	Blood; adult	GPSC9/CC63/ST63	NON-MUCOID	SPRL
SPRLISCIII5174-16	Blood; adult	GPSC98/CC404/ST404	MUCOID	SPRL
SPRLISCIII0930-08	Blood; adult	GPSC98/CC404/ST404	NON-MUCOID	SPRL

Figure S1: Study design and strains used in the different studies. Diagram showing the evolution of IPD by serotype 8 in Spain including the different periods analyzed including 2009–2023 (A). Strains used for WGS analysis were selected from 2008–2021. We included 153 strains from the national collection at ISCIII (42 strains previously sequenced at Hospital Universitari de Bellvitge (HUB; doi: 10.1093/jac/dkac199) and 111 strains sequenced at SPRLISCIII). Strains were selected randomly using the RAND function in Microsoft Excel (2016 [Windows]) to ensure a general distribution from around the country (B). Characteristics of serotype 8 strains used for the different pathogenesis studies (C).

Table S1: Genomes analyzed in the study, accession numbers, metadata and main antibiotic resistance and virulence factors.

			METADATA										
GENOME	ACCESION NUMBER	YEAR	AGE	GPSC	CC	ST	*aroE*	*gdh*	*gki*	*recP*	*spi*	*xpt*	*ddl*
HUB-SP10037	ERS9873913	2009	68	3	53	16359	2	5	1	11	16	3	1070
HUB-SP10933	ERS9873914	2012	32	98	404	404	7	9	15	11	42	1	70
HUB-SP11128	ERS9873915	2012	56	98	404	404	7	9	15	11	42	1	70
HUB-SP11530	ERS9873916	2013	35	3	53	53	2	5	1	11	16	3	14
HUB-SP12039	ERS9873917	2015	57	3	53	53	2	5	1	11	16	3	14
HUB-SP12238	ERS9873918	2015	69	3	53	53	2	5	1	11	16	3	14
HUB-SP12583	ERS9873919	2016	57	3	53	1110	2	5	1	11	16	135	14
HUB-SP13558	ERR13347888	2019	62	3	53	18264	2	5	1	11	16	1096	14
HUB-SP13588	ERR13347890	2019	93	98	404	4960	7	9	15	11	6	1	70
HUB-SP13632	ERR13347897	2019	51	3	53	1110	2	5	1	11	16	135	14
HUB-SP13634	ERR13347898	2019	83	3	53	53	2	5	1	11	16	3	14
HUB-SP13711	ERR13347904	2019	70	3	53	53	2	5	1	11	16	3	14
HUB-SP13723	ERR13347906	2019	54	3	53	53	2	5	1	11	16	3	14
HUB-SP13737	ERR13347907	2019	73	3	53	1110	2	5	1	11	16	135	14
HUB-SP13767B	ERR13347918	2019	71	3	53	1110	2	5	1	11	16	135	14
HUB-SP13807	ERR13347922	2019	59	3	53	53*	2	5	1	truncated	16	135	14
HUB-SP13815	ERR13347923	2019	89	3	53	53	2	5	1	11	16	3	14
HUB-SP13829	ERR13347924	2019	75	98	404	404	7	9	15	11	42	1	70
HUB-SP13830	ERR13347925	2019	44	3	53	1110	2	5	1	11	16	135	14
HUB-SP13844	ERR13347926	2019	54	98	404	404	7	9	15	11	42	1	70
HUB-SP13986	ERR13347932	2019	74	3	53	53	2	5	1	11	16	3	14
HUB-SP14029	ERR13347935	2019	61	3	53	1110	2	5	1	11	16	135	14
HUB-SP14045	ERR13347937	2019	60	3	53	53	2	5	1	11	16	3	14
HUB-SP14052	ERR13347939	2019	64	3	53	1110	2	5	1	11	16	135	14
HUB-SP14053	ERR13347940	2019	84	3	53	53	2	5	1	11	16	3	14
HUB-SP14054	ERR13347941	2019	52	3	53	53	2	5	1	11	16	3	14
HUB-SP14118	ERR13347951	2020	60	98	404	4960	7	9	15	11	6	1	70
HUB-SP14128	ERR13347952	2020	63	98	404	404	7	9	15	11	42	1	70
HUB-SP14252	ERR13347955	2020	74	98	404	404	7	9	15	11	41	1	70
HUB-SP14341	ERR13347964	2020	71	3	53	53	2	5	1	11	16	3	14
HUB-SP14385	ERR13347966	2020	41	3	53	53	2	5	1	11	16	3	14
HUB-SP14412	ERR13347968	2020	54	3	53	53	2	5	1	11	16	3	14
HUB-SP14507	ERR13347971	2020	61	98	404	404	7	9	15	11	42	1	70
HUB-SP14521	ERR13347973	2021	63	3	53	53	2	5	1	11	16	3	14
HUB-SP14549	ERR13347974	2021	65	98	404	404	7	9	15	11	42	1	70
HUB-SP14586	ERR13347976	2021	36	3	53	53	2	5	1	11	16	3	14
HUB-SP14801	ERR13347987	2021	64	3	53	1110	2	5	1	11	16	135	14
HUB-SP14803	ERR13347988	2021	37	3	53	53	2	5	1	11	16	3	14
HUB-SP9445	ERS9873920	2008	24	98	404	404	7	9	15	11	42	1	70
HUB-SP9464	ERS9873921	2008	63	3	53	53	2	5	1	11	16	3	14
HUB-SP9466	ERS9873922	2008	56	98	404	17328	7	9	15	525	42	1	70
HUB-SP9784	ERS9873923	2009	36	3	53	16359	2	5	1	11	16	3	1070
SPRLISCIII0136-20	ERR13906101	2020	18	3	53	1110	2	5	1	11	16	135	14
SPRLISCIII0195-20	ERR13906102	2020	51	3	53	1110	2	5	1	11	16	135	14
SPRLISCIII0210-20	ERR13906103	2020	50	3	53	1110	2	5	1	11	16	135	14
SPRLISCIII0269-20	ERR13906104	2020	60	98	404	404	7	9	15	11	42	1	70
SPRLISCIII0296-20	ERR13906105	2020	60	3	53	53*	2	5	1*	11	16	3	14
SPRLISCIII0342-20	ERR13906106	2020	58	3	53	1110	2	5	1	11	16	135	14
SPRLISCIII0345-20	ERR13906107	2020	42	3	53	1110	2	5	1	11	16	135	14
SPRLISCIII0370-07	ERR13906108	2007	89	9	63	63	2	5	36	12	17	21	14
SPRLISCIII0387-20	ERR13906109	2020	59	3	53	1110	2	5	1	11	16	135	14
SPRLISCIII0421-20	ERR13906110	2020	66	3	53	1110	2	5	1	11	16	135	14
SPRLISCIII0433-20	ERR13906111	2020	21	3	53	53	2	5	1	11	16	3	14
SPRLISCIII0474-20	ERR13906112	2020	80	3	53	53*	2	5	1	11	16	3*	14
SPRLISCIII0679-20	ERR13906113	2020	67	3	53	53	2	5	1	11	16	3	14
SPRLISCIII0696-20	ERR13906114	2020	83	3	53	53	2	5	1	11	16	3	14
SPRLISCIII0785-08	ERR13906115	2008	40	9	63	63	2	5	36	12	17	21	14
SPRLISCIII0849-08	ERR13906116	2008	80	9	63	63	2	5	36	12	17	21	14
SPRLISCIII0930-08	ERR13906117	2008	60	98	404	404	7	9	15	11	42	1	70
SPRLISCIII0973-08	ERR13906118	2008	72	3	53	53	2	5	1	11	16	3	14
SPRLISCIII1088-08	ERR13906119	2008	68	9	63	63	2	5	36	12	17	21	14
SPRLISCIII1229-08	ERR13906120	2008	60	9	63	63	2	5	36	12	17	21	14
SPRLISCIII1383-09	ERR13906121	2009	80	3	53	1110	2	5	1	11	16	135	14
SPRLISCIII1540-09	ERR13906122	2009	49	9	63	63	2	5	36	12	17	21	14
SPRLISCIII1555-09	ERR13906123	2009	41	9	63	63	2	5	36	12	17	21	14
SPRLISCIII1592-09	ERR13906124	2009	75	3	53	53	2	5	1	11	16	3	14

SPRLISCIII2060-09	ERR13906125	2009	53	9	63	63	2	5	36	12	17	21	14
SPRLISCIII2085-09	ERR13906126	2009	51	9	63	63	2	5	36	12	17	21	14
SPRLISCIII2302-10	ERR13906127	2010	75	9	63	63	2	5	36	12	17	21	14
SPRLISCIII2339-10	ERR13906129	2010	41	9	63	63	2	5	36	12	17	21	14
SPRLISCIII2339M1-10	ERR13906128	2010	41	9	63	63	2	5	36	12	17	21	14
SPRLISCIII2409-10	ERR13906130	2010	19	3	53	53	2	5	1	11	16	3	14
SPRLISCIII2746-11	ERR13906131	2011	55	3	53	53	2	5	1	11	16	3	14
SPRLISCIII3021-11	ERR13906132	2011	87	3	53	53	2	5	1	11	16	3	14
SPRLISCIII3056-11	ERR13906133	2011	81	3	53	1110	2	5	1	11	16	135	14
SPRLISCIII3095-19	ERR13906134	2019	62	3	53	1110	2	5	1	11	16	135	14
SPRLISCIII3128-11	ERR13906135	2011	48	9	63	63	2	5	36	12	17	21	14
SPRLISCIII3193-12	ERR13906136	2012	16	3	53	1110	2	5	1	11	16	135	14
SPRLISCIII3371-19	ERR13906137	2019	69	3	53	1110	2	5	1	11	16	135	14
SPRLISCIII3372-12	ERR13906138	2012	77	3	53	1110	2	5	1	11	16	135	14
SPRLISCIII3421-12	ERR13906139	2012	45	9	63	63	2	5	36	12	17	21	14
SPRLISCIII3505-12	ERR13906140	2012	60	3	53	53	2	5	1	11	16	3	14
SPRLISCIII3540-12	ERR13906141	2012	85	9	63	63	2	5	36	12	17	21	14
SPRLISCIII3610-13	ERR13906142	2013	57	3	53	53	2	5	1	11	16	3	14
SPRLISCIII3776-13	ERR13906143	2013	18	3	53	1110	2	5	1	11	16	135	14
SPRLISCIII3832-13	ERR13906144	2013	76	3	53	53	2	5	1	11	16	3	14
SPRLISCIII3859-13	ERR13906145	2013	85	3	53	1110	2	5	1	11	16	135	14
SPRLISCIII4054-14	ERR13906146	2014	81	3	53	1110	2	5	1	11	16	135	14
SPRLISCIII4063-14	ERR13906147	2014	68	3	53	53	2	5	1	11	16	3	14
SPRLISCIII4071-14	ERR13906148	2014	66	3	53	53	2	5	1	11	16	3	14
SPRLISCIII4202-14	ERR13906149	2014	81	9	63	63	2	5	36	12	17	21	14
SPRLISCIII4247-14	ERR13906150	2014	57	3	53	53	2	5	1	11	16	3	14
SPRLISCIII4280-14	ERR13906151	2014	60	3	53	1110	2	5	1	11	16	135	14
SPRLISCIII4285-14	ERR13906152	2014	30	98	404	404	7	9	15	11	42	1	70
SPRLISCIII4411-15	ERR13906153	2015	77	3	53	1110	2	5	1	11	16	135	14
SPRLISCIII4421-15	ERR13906154	2015	72	3	53	53	2	5	1	11	16	3	14
SPRLISCIII4673-15	ERR13906155	2015	84	3	53	1110	2	5	1	11	16	135	14
SPRLISCIII4791-15	ERR13906156	2015	68	3	53	1110	2	5	1	11	16	135	14
SPRLISCIII4977-16	ERR13906157	2016	72	3	53	1110	2	5	1	11	16	135	14
SPRLISCIII4981-16	ERR13906159	2016	60	3	53	1110	2	5	1	11	16	135	14
SPRLISCIII4981M1-16	ERR13906158	2016	60	3	53	1110	2	5	1	11	16	135	14
SPRLISCIII4982-16	ERR13906161	2016	81	3	53	1110	2	5	1	11	16	135	14
SPRLISCIII4982M1-16	ERR13906160	2016	81	3	53	1110	2	5	1	11	16	135	14
SPRLISCIII5027-16	ERR13906162	2016	87	3	53	53	2	5	1	11	16	3	14
SPRLISCIII5029-16	ERR13906163	2016	67	3	53	53	2	5	1	11	16	3	14
SPRLISCIII5031-16	ERR13906164	2016	90	3	53	53	2	5	1	11	16	3	14
SPRLISCIII5038-16	ERR13906165	2016	81	3	53	1110	2	5	1	11	16	135	14
SPRLISCIII5056-16	ERR13906166	2016	61	3	53	53	2	5	1	11	16	3	14
SPRLISCIII5078-16	ERR13906167	2016	54	3	53	1110	2	5	1	11	16	135	14
SPRLISCIII5150-16	ERR13906168	2016	74	3	53	1110	2	5	1	11	16	135	14
SPRLISCIII5174-16	ERR13906169	2016	58	98	404	404	7	9	15	11	42	1	70
SPRLISCIII5178-16	ERR13906170	2016	49	98	404	404	7	9	15	11	42	1	70
SPRLISCIII5221-16	ERR13906172	2016	76	3	53	1110	2	5	1	11	16	135	14
SPRLISCIII5221M1-16	ERR13906171	2016	76	3	53	1110	2	5	1	11	16	135	14
SPRLISCIII5232-16	ERR13906173	2016	80	3	53	1110	2	5	1	11	16	135	14
SPRLISCIII5272-16	ERR13906174	2016	97	3	53	53	2	5	1	11	16	3	14
SPRLISCIII5345-16	ERR13906175	2016	66	3	53	53	2	5	1	11	16	3	14
SPRLISCIII5354-16	ERR13906176	2016	96	3	53	53	2	5	1	11	16	3	14
SPRLISCIII5371-17	ERR13906177	2017	85	3	53	53	2	5	1	11	16	3	14
SPRLISCIII5409-17	ERR13906178	2017	52	3	53	53	2	5	1	11	16	3	14
SPRLISCIII5422-17	ERR13906179	2017	37	3	53	1110	2	5	1	11	16	135	14
SPRLISCIII5462-17	ERR13906180	2017	63	3	53	1110	2	5	1	11	16	135	14
SPRLISCIII5464-17	ERR13906181	2017	56	3	53	1110	2	5	1	11	16	135	14
SPRLISCIII5469-17	ERR13906182	2017	84	3	53	1110	2	5	1	11	16	135	14
SPRLISCIII5491-17	ERR13906183	2017	78	98	404	404	7	9	15	11	42	1	70
SPRLISCIII5517-17	ERR13906184	2017	45	3	53	53	2	5	1	11	16	3	14
SPRLISCIII5796-17	ERR13906185	2017	69	3	53	1110	2	5	1	11	16	135	14
SPRLISCIII5876-17	ERR13906188	2017	69	3	53	53	2	5	1	11	16	3	14
SPRLISCIII5876M1-17	ERR13906186	2017	69	3	53	53	2	5	1	11	16	3	14
SPRLISCIII5876M2-17	ERR13906187	2017	69	3	53	53	2	5	1	11	16	3	14
SPRLISCIII5919-17	ERR13906189	2017	86	3	53	1110	2	5	1	11	16	135	14
SPRLISCIII5945-18	ERR13906190	2018	82	3	53	53	2	5	1	11	16	3	14
SPRLISCIII5995-18	ERR13906191	2018	76	3	53	53	2	5	1	11	16	3	14
SPRLISCIII6003-18	ERR13906192	2018	25	3	53	53	2	5	1	11	16	3	14

SPRLISCIII6005-18	ERR13906193	2018	76	3	53	53	2	5	1	11	16	3	14
SPRLISCIII6011-18	ERR13906194	2018	71	3	53	53	2	5	1	11	16	3	14
SPRLISCIII6014-18	ERR13906195	2018	51	3	53	1110	2	5	1	11	16	135	14
SPRLISCIII6025-18	ERR13906197	2018	80	3	53	1110	2	5	1	11	16	135	14
SPRLISCIII6025M1-18	ERR13906196	2018	80	3	53	1110	2	5	1	11	16	135	14
SPRLISCIII6085-18	ERR13906198	2018	81	9	63	63	2	5	36	12	17	21	14
SPRLISCIII6158-18	ERR13906200	2018	79	3	53	1110	2	5	1	11	16	135	14
SPRLISCIII6158M1-18	ERR13906199	2018	79	3	53	1110	2	5	1	11	16	135	14
SPRLISCIII6253-18	ERR13906202	2018	92	3	53	1110	2	5	1	11	16	135	14
SPRLISCIII6253M1-18	ERR13906201	2018	92	3	53	1110	2	5	1	11	16	135	14
SPRLISCIII6273-18	ERR13906203	2018	57	3	53	53	2	5	1	11	16	3	14
SPRLISCIII6304-18	ERR13906204	2018	65	3	53	53	2	5	1	11	16	3	14
SPRLISCIII6322-18	ERR13906205	2018	84	3	53	1110	2	5	1	11	16	135	14
SPRLISCIII6330-18	ERR13906206	2018	79	3	53	1110	2	5	1	11	16	135	14
SPRLISCIII6342-18	ERR13906207	2018	81	3	53	1110	2	5	1	11	16	135	14
SPRLISCIII6345-18	ERR13906208	2018	71	3	53	53	2	5	1	11	16	3	14
SPRLISCIII6347-18	ERR13906209	2018	90	3	53	53	2	5	1	11	16	3	14
SPRLISCIII6421-18	ERR13906210	2018	83	3	53	1110	2	5	1	11	16	135	14
SPRLISCIII6498-18	ERR13906211	2018	86	3	53	53	2	5	1	11	16	3	14

				ANTIBIOTIC RESISTANCE					
**GENOME**	**PBPtype**	***pbp2b***	***erm*** **(B)**	***tet*** **(M)**	**Mobile genetic element (MGE)**	***folA***	***folP***	***gyrA***	***parC***
HUB-SP10037	3_6_5								
HUB-SP10933	3_2_5	Gly597Glu							
HUB-SP11128	3_2_5	Gly597Glu							
HUB-SP11530	3_6_5								
HUB-SP12039	3_6_5								
HUB-SP12238	3_6_5								
HUB-SP12583	3_6_5								
HUB-SP13558	3_6_5								
HUB-SP13588	3_2_5	Gly597Glu							
HUB-SP13632	3_6_5								
HUB-SP13634	3_6_5								
HUB-SP13711	3_6_5								
HUB-SP13723	3_6_5								
HUB-SP13737	3_6_5								
HUB-SP13767B	3_6_5								
HUB-SP13807	3_6_5								
HUB-SP13815	3_6_5								
HUB-SP13829	3_2_5	Gly597Glu							
HUB-SP13830	3_6_5								
HUB-SP13844	3_2_5	Gly597Glu							
HUB-SP13986	3_6_5								
HUB-SP14029	3_6_5								
HUB-SP14045	3_6_5								
HUB-SP14052	3_6_5								
HUB-SP14053	3_6_5								
HUB-SP14054	3_6_5								
HUB-SP14118	3_2_5	Gly597Glu							
HUB-SP14128	3_2_5	Gly597Glu							
HUB-SP14252	3_2_5	Gly597Glu							
HUB-SP14341	3_6_5								
HUB-SP14385	3_6_5								
HUB-SP14412	3_6_5								
HUB-SP14507	3_2_5	Gly597Glu							
HUB-SP14521	3_6_NEW								
HUB-SP14549	3_2_5	Gly597Glu							
HUB-SP14586	3_6_5								
HUB-SP14801	3_6_5								
HUB-SP14803	3_NEW_05								
HUB-SP9445	3_2_5	Gly597Glu							
HUB-SP9464	3_6_5								
HUB-SP9466	3_2_5	Gly597Glu							
HUB-SP9784	3_6_5								
SPRLISCIII0136-20	3_6_5								
SPRLISCIII0195-20	3_6_5								
SPRLISCIII0210-20	3_6_5								
SPRLISCIII0269-20	3_2_5	Gly597Glu							
SPRLISCIII0296-20	3_6_5								
SPRLISCIII0342-20	3_6_5								
SPRLISCIII0345-20	3_6_5								
SPRLISCIII0370-07	NEW_27_5	Thr446Ala;Leu609Ala;Asp625Gly;Gln628Glu;Thr630Asn	X	X	Tn5252-like	Ile100Leu		Glu85Lys	Ser79Phe
SPRLISCIII0387-20	3_6_5								
SPRLISCIII0421-20	3_6_5								
SPRLISCIII0433-20	3_6_5								
SPRLISCIII0474-20	3_6_5								
SPRLISCIII0679-20	3_6_5								
SPRLISCIII0696-20	3_6_5								
SPRLISCIII0785-08	NEW_27_5	Thr446Ala;Leu609Ala;Asp625Gly;Gln628Glu;Thr630Asn	X	X	Tn5252-like	Ile100Leu		Ser81Phe	Ser79Phe
SPRLISCIII0849-08	NEW_27_5	Thr446Ala;Leu609Ala;Asp625Gly;Gln628Glu;Thr630Asn	X	X	Tn5252-like	Ile100Leu		Ser81Phe	Ser79Phe
SPRLISCIII0930-08	3_2_5	Gly597Glu							
SPRLISCIII0973-08	3_6_5								
SPRLISCIII1088-08	NEW_27_5	Thr446Ala;Leu609Ala;Asp625Gly;Gln628Glu;Thr630Asn	X	X	Tn*5252* -like	Ile100Leu			Ser79Phe
SPRLISCIII1229-08	NEW_27_5	Thr446Ala;Leu609Ala;Asp625Gly;Gln628Glu;Thr630Asn	X	X	Tn*5252* -like	Ile100Leu		Ser81Phe	Ser79Phe
SPRLISCIII1383-09	3_6_5								
SPRLISCIII1540-09	NEW_27_5	Thr446Ala;Leu609Ala;Asp625Gly;Gln628Glu;Thr630Asn	X	X	Tn*5252* -like	Ile100Leu	INSERTION60	Ser81Phe	Ser79Phe
SPRLISCIII1555-09	NEW_27_5	Thr446Ala;Leu609Ala;Asp625Gly;Gln628Glu;Thr630Asn	X	X	Tn*5252* -like	Ile100Leu	INSERTION60	Ser81Phe	Ser79Phe
SPRLISCIII1592-09	3_6_5								
SPRLISCIII2060-09	NEW_27_5	Thr446Ala;Leu609Ala;Asp625Gly;Gln628Glu;Thr630Asn	X	X	Tn*5252* -like	Ile100Leu	INSERTION60	Ser81Phe	Ser79Phe
SPRLISCIII2085-09	NEW_27_5	Thr446Ala;Leu609Ala;Asp625Gly;Gln628Glu;Thr630Asn	X	X	Tn*5252* -like	Ile100Leu		Glu85Lys	Ser79Phe
SPRLISCIII2302-10	NEW_27_5	Thr446Ala;Leu609Ala;Asp625Gly;Gln628Glu;Thr630Asn	X	X	Tn*5252* -like	Ile100Leu			Ser79Phe
SPRLISCIII2339-10	NEW_27_5	Thr446Ala;Leu609Ala;Asp625Gly;Gln628Glu;Thr630Asn	X	X	Tn*5252* -like	Ile100Leu		Ser81Phe	Ser79Phe
SPRLISCIII2339M1-10	NEW_27_5	Thr446Ala;Leu609Ala;Asp625Gly;Gln628Glu;Thr630Asn	X	X	Tn*5252* -like	Ile100Leu		Ser81Phe	Ser79Phe
SPRLISCIII2409-10	3_NEW_5								
SPRLISCIII2746-11	3_6_5								
SPRLISCIII3021-11	3_6_5								
SPRLISCIII3056-11	3_6_5							Ser81Phe; Glu85Lys	Ser79Phe
SPRLISCIII3095-19	3_6_5								
SPRLISCIII3128-11	NEW_27_5	Thr446Ala;Leu609Ala;Asp625Gly;Gln628Glu;Thr630Asn	X	X	Tn*5252* -like	Ile100Leu	INSERTION60	Ser81Phe	Ser79Phe
SPRLISCIII3193-12	3_6_5								
SPRLISCIII3371-19	3_6_5								
SPRLISCIII3372-12	3_6_5								
SPRLISCIII3421-12	NEW_27_5	Thr446Ala;Leu609Ala;Asp625Gly;Gln628Glu;Thr630Asn	X	X	Tn*5252* -like	Ile100Leu	INSERTION60	Ser81Phe	Ser79Phe
SPRLISCIII3505-12	3_6_5								
SPRLISCIII3540-12	NEW_27_5	Thr446Ala;Leu609Ala;Asp625Gly;Gln628Glu;Thr630Asn	X	X	Tn*5252* -like	Ile100Leu	INSERTION60	Ser81Phe	Ser79Phe
SPRLISCIII3610-13	3_6_5								
SPRLISCIII3776-13	3_6_5								
SPRLISCIII3832-13	3_6_5								
SPRLISCIII3859-13	3_6_5								
SPRLISCIII4054-14	3_6_5								
SPRLISCIII4063-14	3_6_5								
SPRLISCIII4071-14	3_6_5								
SPRLISCIII4202-14	NEW_27_5	Thr446Ala;Leu609Ala;Asp625Gly;Gln628Glu;Thr630Asn	X	X	Tn*5252* -like	Ile100Leu		Ser81Phe	Ser79Phe
SPRLISCIII4247-14	3_6_5								
SPRLISCIII4280-14	3_6_5								

SPRLISCIII4285-14	NEW_2_5	Gly597Glu							
SPRLISCIII4411-15	3_6_5								
SPRLISCIII4421-15	3_6_5								
SPRLISCIII4673-15	3_6_5								
SPRLISCIII4791-15	3_6_5								
SPRLISCIII4977-16	3_6_5								
SPRLISCIII4981-16	3_6_5								
SPRLISCIII4981M1-16	3_6_5								
SPRLISCIII4982-16	3_6_5								
SPRLISCIII4982M1-16	3_6_5								
SPRLISCIII5027-16	3_6_5								
SPRLISCIII5029-16	3_6_5								
SPRLISCIII5031-16	3_6_5								
SPRLISCIII5038-16	3_6_5								
SPRLISCIII5056-16	3_6_5								
SPRLISCIII5078-16	3_6_5								
SPRLISCIII5150-16	3_6_5								
SPRLISCIII5174-16	3_2_5	Gly597Glu							
SPRLISCIII5178-16	3_2_5	Gly597Glu							
SPRLISCIII5221-16	3_6_5								
SPRLISCIII5221M1-16	3_6_5								
SPRLISCIII5232-16	3_6_5								
SPRLISCIII5272-16	3_6_5								
SPRLISCIII5345-16	3_6_5								
SPRLISCIII5354-16	3_6_5								
SPRLISCIII5371-17	3_6_5								
SPRLISCIII5409-17	3_6_5								
SPRLISCIII5422-17	3_6_5								
SPRLISCIII5462-17	3_6_5								
SPRLISCIII5464-17	3_6_5								
SPRLISCIII5469-17	3_6_5								
SPRLISCIII5491-17	3_2_5	Gly597Glu							
SPRLISCIII5517-17	3_6_5								
SPRLISCIII5796-17	3_6_5								
SPRLISCIII5876-17	3_6_5								
SPRLISCIII5876M1-17	3_6_5								
SPRLISCIII5876M2-17	3_6_5								
SPRLISCIII5919-17	3_6_5								
SPRLISCIII5945-18	3_6_5								
SPRLISCIII5995-18	3_NEW_5								
SPRLISCIII6003-18	3_6_5								
SPRLISCIII6005-18	3_6_5								
SPRLISCIII6011-18	3_NEW_5								
SPRLISCIII6014-18	3_6_5								
SPRLISCIII6025-18	3_6_5								
SPRLISCIII6025M1-18	3_6_5								
SPRLISCIII6085-18	NEW_27_5	Thr446Ala;Leu609Ala;Asp625Gly;Gln628Glu;Thr630Asn	**X**	**X**	Tn*5252* -like	Ile100Leu	INSERTION60	Ser81Phe	Ser79Phe
SPRLISCIII6158-18	3_6_5							Ser81Phe	Ser79Phe
SPRLISCIII6158M1-18	3_6_5							Ser81Phe	Ser79Phe
SPRLISCIII6253-18	3_6_5								
SPRLISCIII6253M1-18	NEW_6_5								
SPRLISCIII6273-18	3_6_5								
SPRLISCIII6304-18	3_6_5								
SPRLISCIII6322-18	3_6_5								
SPRLISCIII6330-18	3_6_5								
SPRLISCIII6342-18	3_6_5								
SPRLISCIII6345-18	3_6_5								
SPRLISCIII6347-18	3_6_5								
SPRLISCIII6421-18	3_6_5								
SPRLISCIII6498-18	3_NEW_5								

	VIRULENCE FACTORS														
GENOME	*hysA*	*lytA*	ımococcal prophage (	*lytB*	*lytC*	*nanA*	*nanB*	*pavA*	*pce*	*pfbA*	*ply*	*psaA*	*pspA*	*pspC*	*zmpC*
HUB-SP10037	Group 3	20_Spn/FamA		2_Spn	Group 1	Group 2	Group 3	Group 2	Group 3	Group 1	Group 3	Group 1	clade I-4	6.11	Group 1
HUB-SP10933	Group 2		PPH010 + PPH080	2_Spn	Group 2	Group 3		Group 2	Group 2	Asn18*	Group 2	Group 2		6.7	
HUB-SP11128	Group 2	20_Spn/FamA	PPH010	2_Spn	Group 2	Group 3		Group 2	Group 2	Asn18*	Group 2	Group 2	clade IV-1	partial	
HUB-SP11530	Group 3	20_Spn/FamA		partial	Group 1	Group 2	Group 3	Group 2	Group 3	Group 1	Group 3	Group 1	clade I-4	6.11	Group 1
HUB-SP12039	Group 3	20_Spn/FamA		3_Spn	Group 1	Group 2	Group 3	Group 2	Group 3	Group 1	Group 3	Group 1	clade I-4	6.11	Group 1
HUB-SP12238	Group 3	20_Spn/FamA		2_Spn	Group 1	Group 2	Group 3	Group 2	Group 3	Group 1	Group 3	Group 1	clade I-4	6.11	Group 1
HUB-SP12583	Group 3	20_Spn/FamA		2_Spn	Group 1	Group 2	Group 3	Group 2	Group 3	Group 1	Group 3	Group 1	clade I-4	6.11	Group 1
HUB-SP13558	Group 3	20_Spn/FamA		3_Spn	Group 1	Group 2	Group 3	Group 2	Group 3	Group 1	Group 3	Group 1	clade I-4	6.11	Group 1
HUB-SP13588	Group 2	20_Spn/FamA		1_Spn	Group 2	Group 3	Group 1	Group 2	Group 2	Asn18*	Group 2	Group 2	clade IV-1	6.7	
HUB-SP13632	Group 3	20_Spn/FamA		3_Spn	Group 1	Group 2	Group 3	Group 2	Group 3	Group 1	Group 3	Group 1	clade I-4	6.11	Group 1
HUB-SP13634	Group 3	20_Spn/FamA		3_Spn	Group 1	Group 2	Group 3	Group 2	Group 3	Group 1	Group 3	Group 1	clade I-4	6.11	Group 1
HUB-SP13711		20_Spn/FamA		short_lytB	Group 1	Group 2	Group 3	Group 2	Group 3	Group 1	Group 3	Group 1		6.11	Group 1
HUB-SP13723	Group 3	20_Spn/FamA		3_Spn	Group 1	Group 2	Group 3	Group 2	Group 3	Group 1	Group 3	Group 1	clade I-4	6.11	Group 1
HUB-SP13737	Group 3	20_Spn/FamA		3_Spn	Group 1	Group 2	partial	Group 2	Group 3	partial	Group 3	Group 1	clade I-4	6.11	Group 1
HUB-SP13767B	Group 3	20_Spn/FamA		3_Spn	Group 1	Group 2	Group 3	Group 2	Group 3	Group 1	Group 3	Group 1	clade I-4	6.11	Group 1
HUB-SP13807	Group 3	20_Spn/FamA		3_Spn	Group 1	Group 2	Group 3	Group 2	Group 3	Group 1	Group 3	Group 1	clade I-4	6.11	Group 1
HUB-SP13815	Group 3	20_Spn/FamA		3_Spn	Group 1	Group 2	Group 3	Group 2	Group 3	Group 1	Group 3	Group 1	clade I-4	6.11	Group 1
HUB-SP13829	Group 2	20_Spn/FamA		3_Spn	Group 2	Group 3		Group 2	Group 2	Asn18*	Group 2	Group 2	clade IV-1	6.7	
HUB-SP13830	Group 3	20_Spn/FamA		3_Spn	Group 1	Group 2	Group 3	Group 2	Group 3	Group 1	Group 3	Group 1	clade I-4	6.11	Group 1
HUB-SP13844	Group 2	20_Spn/FamA	PPH080	3_Spn	Group 2	Group 3		Group 2	Group 2	Asn18*	Group 2	Group 2	clade IV-1	6.7	
HUB-SP13986		20_Spn/FamA		3_Spn	Group 1	Group 2	Group 3	Group 2	Group 3	Group 1	Group 3	Group 1		6.11	Group 1
HUB-SP14029	Group 3	20_Spn/FamA		3_Spn	Group 1	Group 2	Group 3	Group 2	Group 3	Group 1	Group 3	Group 1	clade I-4	6.11	Group 1
HUB-SP14045	Group 3	20_Spn/FamA		2_Spn	Group 1	Group 2	Group 3	Group 2	Group 3	Group 1	Group 3	Group 1	clade I-4	6.11	Group 1
HUB-SP14052	Group 3	20_Spn/FamA		3_Spn	Group 1	Group 2	Group 3	Group 2	Group 3	Group 1	Group 3	Group 1	clade I-4	6.11	Group 1
HUB-SP14053	Group 3	20_Spn/FamA		3_Spn	Group 1	Group 2	Group 3	Group 2	Group 3	Group 1	Group 3	Group 1	clade I-4	6.11	Group 1
HUB-SP14054	Group 3	20_Spn/FamA		2_Spn	Group 1	Group 2	Group 3	Group 2	Group 3	Group 1	Group 3	Group 1	clade I-4	6.11	Group 1
HUB-SP14118	Group 2	20_Spn/FamA	PPH010	1_Spn	Group 2	Group 3	Group 1	Group 2	Group 2	Asn18*	Group 2	Group 2	clade IV-1	6.7	
HUB-SP14128	Group 2	20_Spn/FamA		3_Spn	Group 2	Group 3		Group 2	Group 2	partial	Group 2	Group 2	clade IV-1	6.7	
HUB-SP14252	Group 2	20_Spn/FamA		3_Spn	Group 2	Group 3		Group 2	Group 2	Asn18*	Group 2	Group 2	clade IV-1	6.7	
HUB-SP14341	Group 3	20_Spn/FamA		3_Spn	Group 1	Group 2	Group 3	Group 2	Group 3	Group 1	Group 3	Group 1	clade I-4	6.11	Group 1
HUB-SP14385	Group 3	20_Spn/FamA		3_Spn	Group 1	Group 2	Group 3	Group 2	Group 3	partial	Group 3	Group 1	clade I-4	6.11	Group 1
HUB-SP14412	Group 3	20_Spn/FamA		3_Spn	Group 1	Group 2	Group 3	Group 2	Group 3	Group 1	Group 3	Group 1	clade I-4	6.11	Group 1
HUB-SP14507	Group 2	20_Spn/FamA +PPH_*lytA*	PPH010	3_Spn	Group 2	Group 3		Group 2	Group 2	Asn18*	Group 2	Group 2	clade IV-1	6.7	
HUB-SP14521	Group 3	20_Spn/FamA		3_Spn	Group 1	Group 2	Group 3	Group 2	Group 3	Group 1	Group 3	Group 1	clade I-4	6.11	Group 1
HUB-SP14549	Group 2	20_Spn/FamA		3_Spn	Group 2	Group 3		Group 2	Group 2	Asn18*	Group 2	Group 2	clade IV-1	6.7	
HUB-SP14586	Group 3	20_Spn/FamA		3_Spn	Group 1	Group 2	Group 3	Group 2	Group 3	Group 1	Group 3	Group 1	clade I-4	6.11	Group 1
HUB-SP14801	Group 3	20_Spn/FamA		3_Spn	Group 1	Group 2	Group 3	Group 2	Group 3	partial	Group 3	Group 1	clade I-4	6.11	Group 1
HUB-SP14803	Group 3	20_Spn/FamA		3_Spn	Group 1	Group 2	Group 3	Group 2	Group 3	Group 1	Group 3	Group 1	clade I-4	6.11	Group 1
HUB-SP9445	Group 2	20_Spn/FamA		partial	Group 2	Group 3		Group 2	Group 2	Asn18*	Group 2	Group 2	clade IV-1	6.7	
HUB-SP9464	Group 3	20_Spn/FamA		2_Spn	Group 1	Group 2	Group 3	Group 2	Group 3	partial	Group 3	Group 1	clade I-4	6.11	Group 1
HUB-SP9466	Group 2	20_Spn/FamA +PPH_*lytA*	PPH010	partial	Group 2	Group 3		Group 2	Group 2	Asn18*	Group 2	Group 2	clade IV-1	6.7	
HUB-SP9784	Group 3	20_Spn/FamA		partial	Group 1	Group 2	Group 3	Group 2	Group 3	Group 1	Group 3	Group 1	clade I-4	6.11	Group 1
SPRLISCIII0136-20	Group 3	20_Spn/FamA		2_Spn	Group 1	Group 2	Group 3	Group 2	Group 3	Group 1	Group 3	Group 1	clade I-4	6.11	Group 1
SPRLISCIII0195-20	Group 3	20_Spn/FamA		partial	Group 1	Group 2	Group 3	Group 2	Group 3	Group 1	Group 3	Group 1	clade I-4	6.11	Group 1
SPRLISCIII0210-20	Group 3	20_Spn/FamA		partial	Group 1	Group 2	Group 3	Group 2	Group 3	Group 1	Group 3	Group 1	clade I-4	6.11	Other
SPRLISCIII0269-20	Group 2	20_Spn/FamA + 2 PPH_*lytA*	PPH100 + PPH080	partial	Group 2	Group 3		Group 2	Group 2	Asn18*	Group 2	Group 2	clade IV-1	partial	
SPRLISCIII0296-20	Group 3	20_Spn/FamA		2_Spn	Group 1	Group 2	Group 3	Group 2	Group 3	Group 1	Group 3	Group 1	clade I-4	6.11	Group 1
SPRLISCIII0342-20	Group 3	20_Spn/FamA		2_Spn	Group 1	Group 2	Group 3	Group 2	Group 3	Group 1	Group 3	Group 1	clade I-4	6.11	Group 1
SPRLISCIII0345-20	Group 3	20_Spn/FamA		partial	Group 1	Group 2	Group 3	Group 2	Group 3	Group 1	Group 3	Group 1	clade I-4	6.11	Group 1
SPRLISCIII0370-07	Group 1 (inserted Ser89)	23_Spn/FamA		1_Spn	Group 3	Group 1	Group 2	Group 1	Group 1	Group 2	Group 1	Group 2	clade IV-2	7.1	Group 3
SPRLISCIII0387-20	Group 3	20_Spn/FamA		2_Spn	Group 1	Group 2	Group 3	Group 2	Group 3	Group 1	Group 3	Group 1	clade I-4	6.11	Group 1
SPRLISCIII0421-20	Group 3	20_Spn/FamA		partial	Group 1	Group 2	Group 3	Group 2	Group 3	Group 1	Group 3	Group 1	clade I-4	6.11	Group 1
SPRLISCIII0433-20	Group 3	20_Spn/FamA		2_Spn	Group 1	Group 2	Group 3	Group 2	Group 3	Group 1	Group 3	Group 1	clade I-4	6.11	Group 1
SPRLISCIII0474-20	Group 3	20_Spn/FamA		partial	Group 1	Group 2	Group 3	Group 2	Group 3	Group 1	Group 3	Group 1	clade I-4	6.11	Other
SPRLISCIII0679-20	Group 3	20_Spn/FamA		2_Spn	Group 1	Group 2	Group 3	Group 2	Group 3	Group 1	Group 3	Group 1		6.11	Group 1
SPRLISCIII0696-20	Group 3	20_Spn/FamA		partial	Group 1	Group 2	Group 3	Group 2	Group 3	Group 1	Group 3	Group 1	clade I-4	6.11	Group 1
SPRLISCIII0785-08	Group 1 (inserted Ser89)	23_Spn/FamA		1_Spn	Group 1	Group 1	Group 2	Group 1	Group 1	Group 2	Group 1	Group 2	clade IV-2	7.1	Group 3
SPRLISCIII0849-08	Group 1 (inserted Ser89)	23_Spn/FamA		1_Spn	Group 1	Group 1	Group 2	Group 1	Group 1	Group 2	Group 1	Group 2		7.1	Group 3
SPRLISCIII0930-08	Group 2	20_Spn/FamA +PPH_*lytA*	PPH010	3_Spn	Group 2	Group 3		Group 2	Group 2	Asn18*	Group 2	Group 2	clade IV-1	partial	
SPRLISCIII0973-08	Group 3	20_Spn/FamA		3_Spn	Group 1	Group 2	Group 3	Group 2	Group 3	Group 1	Group 3	Group 1	clade I-4	6.11	Group 1
SPRLISCIII1088-08	Group 1 (inserted Ser89)	23_Spn/FamA		1_Spn	Group 1	Group 1	Group 2	Group 1	Group 1	Group 2	Group 1	Group 2	clade IV-2	7.1	Group 3
SPRLISCIII1229-08	Group 1 (inserted Ser89)	23_Spn/FamA		1_Spn	Group 1	Group 1	Group 2	Group 1	Group 1	Group 2	Group 1	Group 2	clade IV-2	7.1	Group 3
SPRLISCIII1383-09	Group 3	20_Spn/FamA		partial	Group 1	Group 2	Group 3	Group 2	Group 3	Group 1	Group 3	Group 1	clade I-4	6.11	Group 1
SPRLISCIII1540-09	Group 1 (inserted Ser89)	23_Spn/FamA		1_Spn	Group 1	Group 1	Group 2	Group 1	Group 1	Group 2	Group 1	Group 2		7.1	Group 3
SPRLISCIII1555-09	Group 1 (inserted Ser89)	23_Spn/FamA		1_Spn	Group 1	Group 1	Group 2	Group 1	Group 1	Group 2	Group 1	Group 2		7.1	Group 3
SPRLISCIII1592-09	Group 3	20_Spn/FamA		other allele	Group 1	Group 2	Group 3	Group 2	Group 3	Group 1	Group 3	Group 1	clade I-4	6.11	Group 1
SPRLISCIII2060-09	Group 1 (inserted Ser89)	23_Spn/FamA		1_Spn	Group 1	Group 1	Group 2	Group 1	Group 1	Group 2	Group 1	Group 2		7.1	Group 3
SPRLISCIII2085-09	Group 1 (inserted Ser89)	23_Spn/FamA		1_Spn	Group 1	Group 1	Group 2	Group 1	Group 1	Group 2	Group 1	Group 2	clade IV-2	7.1	Group 3
SPRLISCIII2302-10	Group 1 (inserted Ser89)	23_Spn/FamA		1_Spn	Group 3	Group 1	Group 2	Group 1	Group 1	Group 2	Group 1	Group 2	clade IV-2	7.1	Group 3
SPRLISCIII2339-10	Group 1 (inserted Ser89)	23_Spn/FamA		1_Spn	Group 1	Group 1	Group 2	Group 1	Group 1	Group 2	Group 1	Group 2		7.1	Group 3
SPRLISCIII2339M1-10	Group 1 (inserted Ser89)	23_Spn/FamA		1_Spn	Group 1	Group 1	Group 2	Group 1	Group 1	Group 2	Group 1	Group 2		7.1	Group 3
SPRLISCIII2409-10	Group 3	20_Spn/FamA		partial	Group 1	Group 2	Group 3	Group 2	Group 3	Group 1	Group 3	Group 1	clade I-4	6.11	Group 1
SPRLISCIII2746-11	Group 3	20_Spn/FamA		partial	Group 1	Group 2	Group 3	Group 2	Group 3	Group 1	Group 3	Group 1	clade I-4	6.11	Group 1
SPRLISCIII3021-11	Group 3	20_Spn/FamA		partial	Group 1	Group 2	Group 3	Group 2	Group 3	Group 1	Group 3	Group 1	clade I-4	6.11	Group 1
SPRLISCIII3056-11	Group 3	20_Spn/FamA		partial	Group 1	Group 2	Group 3	Group 2	Group 3	Group 1	Group 3	Group 1	clade I-4	6.11	Group 1
SPRLISCIII3095-19	Group 3	20_Spn/FamA		2_Spn	Group 1	Group 2	Group 3	Group 2	Group 3	Group 1	Group 3	Group 1	clade I-4	6.11	Group 1
SPRLISCIII3128-11	Group 1 (inserted Ser89)	23_Spn/FamA		1_Spn	Group 1	Group 1	Group 2	Group 1	Group 1	Group 2	Group 1	Group 2	clade IV-2	truncated	Group 3
SPRLISCIII3193-12	Group 3	20_Spn/FamA		partial	Group 1	Group 2	Group 3	Group 2	Group 3	Group 1	Group 3	Group 1	clade I-4	6.11	Group 1
SPRLISCIII3371-19	Group 3	20_Spn/FamA		partial	Group 1	Group 2	Group 3	Group 2	Group 3	Group 1	Group 3	Group 1	clade I-4	6.11	Group 1
SPRLISCIII3372-12	Group 3	20_Spn/FamA		partial	Group 1	Group 2	Group 3	Group 2	Group 3	Group 1	Group 3	Group 1	clade I-4	6.11	Group 1
SPRLISCIII3421-12	Group 1 (inserted Ser89)	23_Spn/FamA		1_Spn	Group 1	Group 1	Group 2	Group 1	Group 1	Group 2	Group 1	Group 2	clade IV-2	truncated	Group 3
SPRLISCIII3505-12	Group 3	20_Spn/FamA		3_Spn	Group 1	Group 2	Group 3	Group 2	Group 3	Group 1	Group 3	Group 1	clade I-4	6.11	Group 1
SPRLISCIII3540-12	Group 1 (inserted Ser89)	23_Spn/FamA		1_Spn	Group 1	Group 1	Group 2	Group 1	Group 1	Group 2	Group 1	Group 2	clade IV-2	7.1	Group 3
SPRLISCIII3610-13	Group 3	20_Spn/FamA		2_Spn	Group 1	Group 2	Group 3	Group 2	Group 3	Group 1	Group 3	Group 1	clade I-4	6.11	Group 1
SPRLISCIII3776-13	Group 3	20_Spn/FamA		2_Spn	Group 1	Group 2	Group 3	Group 2	Group 3	Group 1	Group 3	Group 1	clade I-4	6.11	Group 1
SPRLISCIII3832-13	Group 3	20_Spn/FamA		partial	Group 1	Group 2	Group 3	Group 2	Group 3	Group 1	Group 3	Group 1	clade I-4	6.11	Group 1
SPRLISCIII3859-13	Group 3	20_Spn/FamA		2_Spn	Group 1	Group 2	Group 3	Group 2	Group 3	Group 1	Group 3	Group 1	clade I-4	6.11	Group 1
SPRLISCIII4054-14	Group 3	20_Spn/FamA		2_Spn	Group 1	Group 2	Group 3	Group 2	Group 3	Group 1	Group 3	Group 1	clade I-4	6.11	Group 1
SPRLISCIII4063-14	Group 3	20_Spn/FamA		partial	Group 1	Group 2	Group 3	Group 2	Group 3	Group 1	Group 3	Group 1	clade I-4	6.11	Group 1
SPRLISCIII4071-14	Group 3	20_Spn/FamA		partial	Group 1	Group 2	Group 3	Group 2	Group 3	Group 1	Group 3	Group 1	clade I-4	6.11	Gln1079*
SPRLISCIII4202-14	Group 1 (inserted Ser89)	23_Spn/FamA		1_Spn	Group 1	Group 1	Group 2	Group 1	Group 1	Group 2	Group 1	Group 2		7.1	Group 3
SPRLISCIII4247-14	Group 3	20_Spn/FamA		3_Spn	Group 1	Group 2	Group 3	Group 2	Group 3	Group 1	Group 3	Group 1	clade I-4	6.11	Group 1
SPRLISCIII4280-14	Group 3	20_Spn/FamA		3_Spn	Group 1	Group 2	Group 3	Group 2	Group 3	Group 1	Group 3	Group 1	clade I-4	6.11	Group 1
SPRLISCIII4285-14	Group 2	20_Spn/FamA +PPH_*lytA*	PPH010	partial	Group 2	Group 3		Group 2	Group 2	Asn18*	Group 2	Group 2	clade IV-1	6.7	
SPRLISCIII4411-15	Group 3	20_Spn/FamA		partial	Group 1	Group 2	Group 3	Group 2	Group 3	Group 1	Group 3	Group 1	clade I-4	6.11	Group 1
SPRLISCIII4421-15	Group 3	20_Spn/FamA		partial	Group 1	Group 2	Group 3	Group 2	Group 3	Group 1	Group 3	Group 1	clade I-4	6.11	Group 1
SPRLISCIII4673-15	Group 3	20_Spn/FamA		partial	Group 1	Group 2	Group 3	Group 2	Group 3	Group 1	Group 3	Group 1	clade I-4	6.11	Other
SPRLISCIII4791-15	Group 3	20_Spn/FamA		partial	Group 1	Group 2	Group 3	Group 2	Group 3	Group 1	Group 3	Group 1	clade I-4	6.11	Group 1
SPRLISCIII4977-16	Group 3	20_Spn/FamA		3_Spn	Group 1	Group 2	Group 3	Group 2	Group 3	Group 1	Group 3	Group 1	clade I-4	6.11	Group 2
SPRLISCIII4981-16	Group 3	20_Spn/FamA		3_Spn	Group 1	Group 2	Group 3	Group 2	Group 3	Group 1	Group 3	Group 1	clade I-4	6.11	Group 1
SPRLISCIII4981M1-16	Group 3	20_Spn/FamA		partial	Group 1	Group 2	Group 3	Group 2	Group 3	Group 1	Group 3	Group 1	clade I-4	6.11	Group 1
SPRLISCIII4982-16	Group 3	20_Spn/FamA		partial	Group 1	Group 2	Group 3	Group 2	Group 3	Group 1	Group 3	Group 1	clade I-4	6.11	Group 2
SPRLISCIII4982M1-16	Group 3	20_Spn/FamA		partial	Group 1	Group 2	Group 3	Group 2	Group 3	Group 1	Group 3	Group 1	clade I-4	6.11	Group 2
SPRLISCIII5027-16	Group 3	20_Spn/FamA		partial	Group 1	Group 2	Group 3	Group 2	Group 3	Group 1	Group 3	Group 1	clade I-4	6.11	Group 1

SPRLISCIII5029-16	Group 3	20_Spn/FamA		3_Spn	Group 1	Group 2	Group 3	Group 2	Group 3	Group 1	Group 3	Group 1		6.11	Group 1
SPRLISCIII5031-16	Group 3	20_Spn/FamA		partial	Group 1	Group 2	Group 3	Group 2	Group 3	Group 1	Group 3	Group 1	clade I-4	6.11	Group 1
SPRLISCIII5038-16	Group 3	20_Spn/FamA		3_Spn	Group 1	Group 2	Group 3	Group 2	Group 3	Group 1	Group 3	Group 1	clade I-4	6.11	Group 1
SPRLISCIII5056-16	Group 3	20_Spn/FamA		3_Spn	Group 1	Group 2	Group 3	Group 2	Group 3	Group 1	Group 3	Group 1	clade I-4	6.11	Group 1
SPRLISCIII5078-16	Group 3	20_Spn/FamA		3_Spn	Group 1	Group 2	Group 3	Group 2	Group 3	Group 1	Group 3	Group 1	clade I-4	6.11	Group 1
SPRLISCIII5150-16	Group 3	20_Spn/FamA +PPH_*lytA*	PPH085	2_Spn	Group 1	Group 2	Group 3	Group 2	Group 3	Group 1	Group 3	Group 1	clade I-4	6.11	Group 1
SPRLISCIII5174-16	Group 2	20_Spn/FamA +PPH_*lytA*	PPH010	2_Spn	Group 2	Group 3		Group 2	Group 2	Asn18*	Group 2	Group 2	clade IV-1	6.7	
SPRLISCIII5178-16	Group 2	20_Spn/FamA		partial	Group 2	Group 3		Group 2	Leu293*	Asn18*	Group 2	Group 2	clade IV-1	6.7	
SPRLISCIII5221-16	Group 3	20_Spn/FamA		partial	Group 1	Group 2	Group 3	Group 2	Group 3	Group 1	Group 3	Group 1	clade I-4	6.11	Group 1
SPRLISCIII5221M1-16	Group 3	20_Spn/FamA		partial	Group 1	Group 2	Group 3	Group 2	Group 3	Group 1	Group 3	Group 1	clade I-4	6.11	Group 1
SPRLISCIII5232-16	Group 3	20_Spn/FamA		3_Spn	Group 1	Group 2	Group 3	Group 2	Group 3	Group 1	Group 3	Group 1	clade I-4	6.11	Group 1
SPRLISCIII5272-16	Group 3	20_Spn/FamA		partial	Group 1	Group 2	Group 3	Group 2	Group 3	Group 1	Group 3	Group 1	clade I-4	6.11	Group 1
SPRLISCIII5345-16	Group 3	20_Spn/FamA		partial	Group 1	Group 2	Group 3	Group 2	Group 3	Group 1	Group 3	Group 1		6.11	Group 1
SPRLISCIII5354-16	Group 3	20_Spn/FamA		partial	Group 1	Group 2	Group 3	Group 2	Group 3	Group 1	Group 3	Group 1	clade I-4	6.11	Group 1
SPRLISCIII5371-17	Group 3	20_Spn/FamA		partial	Group 1	Group 2	Group 3	Group 2	Group 3	Group 1	Group 3	Group 1	clade I-4	6.11	Group 1
SPRLISCIII5409-17	Group 3	20_Spn/FamA		2_Spn	Group 1	Group 2	Group 3	Group 2	Group 3	Group 1	Group 3	Group 1	clade I-4	6.11	Group 1
SPRLISCIII5422-17	Group 3	20_Spn/FamA		partial	Group 1	Group 2	Group 3	Group 2	Group 3	Group 1	Group 3	Group 1	clade I-4	6.11	Group 1
SPRLISCIII5462-17	Group 3	20_Spn/FamA		partial	Group 1	Group 2	Group 3	Group 2	Group 3	Group 1	Group 3	Group 1	clade I-4	6.11	Group 1
SPRLISCIII5464-17	Group 3	20_Spn/FamA		partial	Group 1	Group 2	Group 3	Group 2	Group 3	Group 1	Group 3	Group 1	clade I-4	6.11	Group 1
SPRLISCIII5469-17	Group 3	20_Spn/FamA		partial	Group 1	Group 2	Group 3	Group 2	Group 3	Group 1	Group 3	Group 1	clade I-4	6.11	Group 1
SPRLISCIII5491-17	Group 2	20_Spn/FamA +PPH_*lytA*	PPH010	3_Spn	Group 2	Group 3		Group 2	Group 2	Asn18*	Group 2	Group 2	clade IV-1	6.7	
SPRLISCIII5517-17	Group 3	20_Spn/FamA		partial	Group 1	Group 2	Group 3	Group 2	Group 3	Group 1	Group 3	Group 1	clade I-4	6.11	Group 1
SPRLISCIII5796-17	Group 3	20_Spn/FamA		3_Spn	Group 1	Group 2	Group 3	Group 2	Group 3	Group 1	Group 3	Group 1	clade I-4	6.11	Group 1
SPRLISCIII5876-17	Group 3	20_Spn/FamA		partial	Group 1	Group 2	Group 3	Group 2	Group 3	Group 1	Group 3	Group 1	clade I-4	6.11	Group 1
SPRLISCIII5876M1-17	Group 3	20_Spn/FamA		2_Spn	Group 1	Group 2	Group 3	Group 2	Group 3	Group 1	Group 3	Group 1	clade I-4	6.11	Group 1
SPRLISCIII5876M2-17	Group 3	20_Spn/FamA		partial	Group 1	Group 2	Group 3	Group 2	Group 3	Group 1	Group 3	Group 1	clade I-4	6.11	Group 1
SPRLISCIII5919-17	Group 3	20_Spn/FamA		3_Spn	Group 1	Group 2	Group 3	Group 2	Group 3	Group 1	Group 3	Group 1	clade I-4	6.11	Group 1
SPRLISCIII5945-18	Group 3	20_Spn/FamA		partial	Group 1	Group 2	Group 3	Group 2	Group 3	Group 1	Group 3	Group 1	clade I-4	6.11	Group 1
SPRLISCIII5995-18	Group 3	20_Spn/FamA		partial	Group 1	Group 2	Group 3	Group 2	Group 3	Group 1	Group 3	Group 1	clade I-4	6.11	Group 1
SPRLISCIII6003-18	Group 3	20_Spn/FamA		3_Spn	Group 1	Group 2	Group 3	Group 2	Group 3	Group 1	Group 3	Group 1	clade I-4	6.11	Group 1
SPRLISCIII6005-18	Group 3	20_Spn/FamA		partial	Group 1	Group 2	Group 3	Group 2	Group 3	Group 1	Group 3	Group 1	clade I-4	6.11	Group 1
SPRLISCIII6011-18	Group 3	20_Spn/FamA		partial	Group 1	Group 2	Group 3	Group 2	Group 3	Group 1	Group 3	Group 1	clade I-4	6.11	Group 1
SPRLISCIII6014-18	Group 3	20_Spn/FamA		partial	Group 1	Group 2	Group 3	Group 2	Group 3	Group 1	Group 3	Group 1	clade I-4	6.11	Group 1
SPRLISCIII6025-18	Group 3	20_Spn/FamA		partial	Group 1	Group 2	Group 3	Group 2	Group 3	Group 1	Group 3	Group 1	clade I-4	6.11	Group 1
SPRLISCIII6025M1-18	Group 3	20_Spn/FamA		partial	Group 1	Group 2	Group 3	Group 2	Group 3	Group 1	Group 3	Group 1	clade I-4	6.11	Group 1
SPRLISCIII6085-18	Group 1 (inserted Ser89)	23_Spn/FamA		1_Spn	Group 1	Group 1	Group 2	Group 1	Group 1	Group 2	Group 1	Group 2		truncated	Group 3
SPRLISCIII6158-18	Group 3	20_Spn/FamA		3_Spn	Group 1	Group 2	Group 3	Group 2	Group 3	Group 1	Group 3	Group 1	clade I-4	6.11	Group 1
SPRLISCIII6158M1-18	Group 3	20_Spn/FamA		partial	Group 1	Group 2	Group 3	Group 2	Group 3	Group 1	Group 3	Group 1	clade I-4	6.11	Group 1
SPRLISCIII6253-18	Group 3	20_Spn/FamA		3_Spn	Group 1	Group 2	Group 3	Group 2	Group 3	Group 1	Group 3	Group 1	clade I-4	6.11	Group 1
SPRLISCIII6253M1-18	Group 3	20_Spn/FamA		partial	Group 1	Group 2	Group 3	Group 2	Group 3	Group 1	Group 3	Group 1	clade I-4	6.11	Group 1
SPRLISCIII6273-18	Group 3	20_Spn/FamA		partial	Group 1	Group 2	Group 3	Group 2	Group 3	Group 1	Group 3	Group 1	clade I-4	6.11	Group 1
SPRLISCIII6304-18	Group 3	20_Spn/FamA		partial	Group 1	Group 2	Group 3	Group 2	Group 3	Group 1	Group 3	Group 1	clade I-4	6.11	Group 1
SPRLISCIII6322-18	Group 3	20_Spn/FamA		partial	Group 1	Group 2	Group 3	Group 2	Group 3	Group 1	Group 3	Group 1	clade I-4	6.11	Group 1
SPRLISCIII6330-18	Group 3	20_Spn/FamA		partial	Group 1	Group 2	Group 3	Group 2	Group 3	Group 1	Group 3	Group 1	clade I-4	6.11	Group 1
SPRLISCIII6342-18	Group 3	20_Spn/FamA		partial	Group 1	Group 2	Group 3	Group 2	Group 3	Group 1	Group 3	Group 1	clade I-4	6.11	Group 1
SPRLISCIII6345-18	Group 3	20_Spn/FamA		partial	Group 1	Group 2	Group 3	Group 2	Group 3	Group 1	Group 3	Group 1	clade I-4	6.11	Group 1
SPRLISCIII6347-18	Group 3	20_Spn/FamA		partial	Group 1	Group 2	Group 3	Group 2	Group 3	Group 1	Group 3	Group 1	clade I-4	6.11	Group 1
SPRLISCIII6421-18	Group 3	20_Spn/FamA		partial	Group 1	Group 2	Group 3	Group 2	Group 3	Group 1	Group 3	Group 1	clade I-4	6.11	Group 1
SPRLISCIII6498-18	Group 3	20_Spn/FamA		partial	Group 1	Group 2	Group 3	Group 2	Group 3	Group 1	Group 3	Group 1	clade I-4	6.11	Group 1

Table S2: Bioinformatic workflow used for WGS analysis.

Workflow steps	Tool	Reference
Read quality control (FASTQC), removal of low-quality sequences (Trimmomatic), elimination of possible contaminations from other bacterial species, and *de novo* assembly of reads (SPAdes)	INNUca	MP Machado, J Halkilahti, A Jaakkonen, DN Silva, I Mendes, Y Nalbantoglu, V Borges, M Ramirez, M Rossi, JA Carriço. INNUca GitHub https://github.com/B-UMMI/INNUca
MLST profile	Mlst	Seemann T. mlst Github https://github.com/tseemann/mlst
Global contextualization with lineage assignation	PathogenWatch	Version 22.5.5. https://pathogen.watch/
Antimicrobial resistance and virulence factor profiling	Abricate (Databases: ncbi, card, argannot, resfinder, vfdb)	Seemann T. Abricate Github https://github.com/tseemann/abricate
PBP typing	Pbptyper	Petit III RA. pbptyper: In silico Penicillin Binding Protein (PBP) typer for *Streptococcus pneumoniae* assemblies Github https://github.com/rpetit3/pbptyper
Genome and pan-genome analysis, comparison of genes between different lineages	Prokka, Roary and roProfile	Seemann T. Prokka: rapid prokaryotic genome annotation. *Bioinformatics* 2014 15;30(14):2068-9. https://github.com/tseemann/prokka; Andrew J. Page, Carla A. Cummins, Martin Hunt, Vanessa K. Wong, Sandra Reuter, Matthew T. G. Holden, Maria Fookes, Daniel Falush, Jacqueline A. Keane, Julian Parkhill. Roary: Rapid large-scale prokaryote pan genome analysis. *Bioinformatics* 2015 31(22):3691-3693. https://sanger-pathogens.github.io/Roary/; roProfile https://github.com/cimendes/roProfile.
Single nucleotide polymorphism (SNP) analysis, construction of phylogenetic trees and genome-wide prediction of recombination	Snippy, RaxML-NG and Gubbins	Seemann T. Snippy Github https://github.com/tseemann/snippy ; Stamatakis A. RAxML version 8: a tool for phylogenetic analysis and post-analysis of large phylogenies. *Bioinformatics* 2014 30(9): 1312-1313. https://github.com/amkozlov/raxml-ng; Croucher N. J., Page A. J., Connor T. R., Delaney A. J., Keane J. A., Bentley S. D., Parkhill J., Harris S.R. Rapid phylogenetic analysis of large samples of recombinant bacterial whole genome sequences using Gubbins. *Nucleic Acids Research* 2014 18;43(3):e15. https://github.com/nickjcroucher/gubbins
Search of mobile genetic elements (MGEs)	ICEberg 3.0	M. Wang, G Liu, M Liu, C Tai, Z. Deng, J Song, H.Y. Ou. ICEberg 3.0: functional categorization and analysis of the integrative and conjugative elements in bacteria. *Nucleic Acids Research* 2024 5; 52(D1):D732-D737. https://tool2-mml.sjtu.edu.cn/ICEberg3/

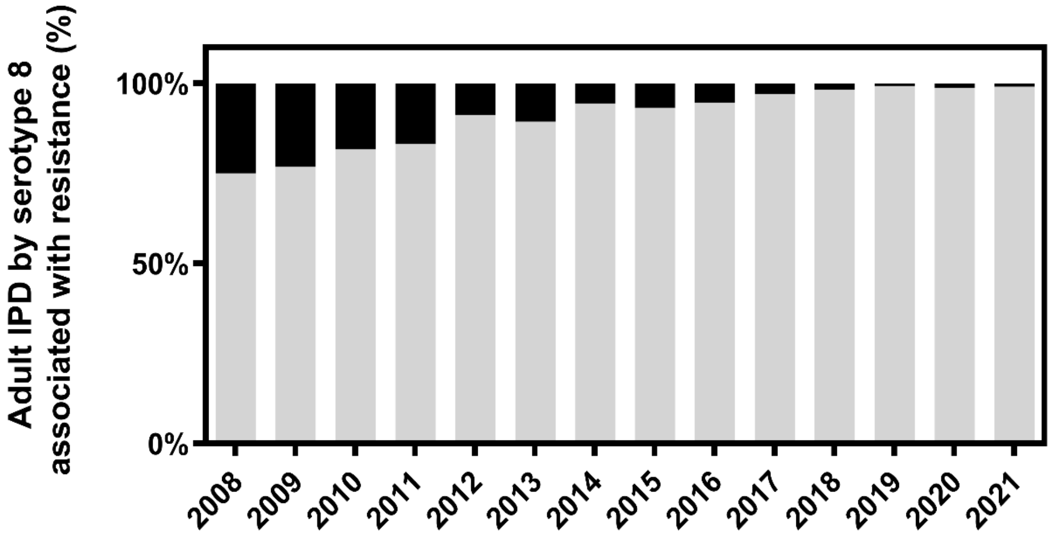

Figure S2: Evolution of serotype 8 strains harboring multidrug resistance (MDR) to erythromycin, clindamycin and tetracycline during 2008-2021. MDR strains are represented in black whereas susceptible strains are represented in grey. The MDR phenotype was associated with CC63/GPSC9.

Table S3: Main differences in virulence factors between serotype 8 genetic lineages.

	**Alleles and % of isolates containing them**		
**Virulence factor**	**GPSC3**	**GPSC98**	**GPSC9**
hysA	Group 3 (100%)	Group 2 (100%)	Group 1 (100%)
lytA	20_Spn/FamA (99.15%)	20_Spn/FamA + PPH_*lytA* or 2 (44.44%)	23_Spn/FamA (100%)
lytB	3_Spn (31.36%)	3_Spn (44.44%)	1_Spn (100%)
lytC	Group 1 (100%)	Group 1 (88.24%)	Group 2 (100%)
nanA	Group 2 (100%)	Group 3 (100%)	Group 1 (100%)
nanB	Group 3 (100%)	Absent (88.89%)	Group 2 (100%)
pavA	Group 2 (100%)	Group 2 (100%)	Group 1 (100%)
pce	Group 3 (100%)	Group 2 (94.44%)	Group 1 (100%)
pfbA	Group 1 (96.61%)	Truncated_Asn18* (100%)	Group 2 (100%)
ply	Group 3 (100%)	Group 2 (100%)	Group 1 (100%)
psaA	Group 1 (100%)	Group 2 (100%)	Group 2 (100%)
pspA	Clade I-4 (95.76%)	Clade IV-2 (52.94%)	Clade IV-1 (94.44%)
pspC	6.11 (100%)	6.7 (83.33%)	7.1 (77.78%)
zmpC	Group 1 (94.07%)	Absent (100%)	Group 3 (100%)

Table S4: Mucoid phenotype study showing polymorphisms that produce changes in proteins of the capsular operon.

				dexB-tnp region		spacing sequence		*wzg*	*wzd*			*wze*					*wchA*												*wciQ*			*wciR*			*wciS*			*wzx*					*wzy*	*upg*			
				-1318	-1255			1352	2414	2599	2993	3012	3089	3128	3170	3341	4115	4237	4271	4329	4336	4424	4447	4585	4604	4679	4796	4809	4982	5003	5004	5482	5608	5619	6284	6855	6932	7445	7573	7854	7857	8300	10227	11094	11308	11409	11640
GENOME	PHENOTYPE	POLYMORPHISM (MISSENSE, NONSENSE, DELETIONS, INSERTIONS)	CHANGE (PROTEIN)	A1318	C1255	-169 (G)	-63(-)	T	A	G	A	G	C	A	T	G	G	A	T	T	T	C	G	C	C	C	G	A	G	G	C	G	G	T	C	C	C	A	C	C	C	T	A	C	C	C	G
SPRLISCIII0195-20	NON-MUCOID	YES	Wzx(Ala164Val)	.	T	.	-	C	.	.	.	.	.	.	.	C	.	.	.	.	.	.	.	.	.	.	.	.	.	.	.	.	.	.	.	.	.	.	T	.	.	C	.	.	.	G	.
SPRLISCIII0370-07	NON-MUCOID	YES	WchA(Ala261Asp)	.	T	.	A	C	.	.	.	.	.	.	.	C	.	.	.	.	.	A	.	.	.	.	.	.	.	.	.	.	.	.	.	.	.	.	.	.	.	.	.	.	.	.	.
SPRLISCIII0421-20	NON-MUCOID	YES	Wze(Thr52Ile);Wcha(Ala454Val)	.	T	.	-	C	.	.	.	.	T	.	.	C	.	.	.	.	.	.	.	.	.	.	.	.	.	.	T	.	.	.	.	.	.	.	.	.	.	C	.	.	.	.	.
SPRLISCIII0679-20	NON-MUCOID	NO		.	T	.	-	C	.	.	.	.	.	.	.	C	.	.	.	.	.	.	.	.	.	.	.	.	.	.	.	.	.	.	.	.	.	.	.	.	.	C	.	.	.	.	.
SPRLISCIII0930-08	NON-MUCOID	YES	WchA(Arg315Cys)	.	.	A	-	C	.	.	.	.	.	.	.	.	.	.	.	.	.	.	.	T	.	.	.	.	.	.	.	.	.	.	.	.	.	G	.	.	.	.	.	.	.	.	.
SPRLISCIII2339-10	NON-MUCOID	YES	WchA(Arg315Cys)	.	T	.	A	C	.	.	.	.	.	.	.	C	.	.	.	.	.	.	.	T	.	.	.	.	.	.	.	.	.	.	.	.	.	.	.	.	.	.	.	.	.	.	.
SPRLISCIII3056-11	NON-MUCOID	YES	Wze(Met36Ile); WchA(Gly158Glu,Asn199Asp,385*)	.	T	.	-	C	.	.	.	C	.	.	.	C	A	G	.	.	.	.	.	.	.	.	T	.	.	.	.	.	.	.	.	.	.	.	.	.	.	C	.	.	.	.	.
SPRLISCIII3505-12	NON-MUCOID	YES	Wzg truncated	.	T	.	-	C	.	.	.	.	.	.	.	C	.	.	.	.	.	.	.	.	.	.	.	.	.	.	.	.	.	.	.	.	.	.	.	.	.	C	.	.	.	.	.
SPRLISCIII4202-14	NON-MUCOID	YES	WciQ(Gly156Trp)	.	T	.	A	C	.	.	.	.	.	.	.	C	.	.	.	.	.	.	.	.	.	.	.	.	.	.	.	T	.	.	.	.	.	.	.	.	.	.	.	.	.	.	.
SPRLISCIII4280-14	NON-MUCOID	YES	WchA(Tyr389Ser)	.	T	.	-	C	.	.	.	.	.	.	.	C	.	.	.	.	.	.	.	.	.	.	.	C	.	.	.	.	.	.	.	.	.	.	.	.	.	C	.	.	.	.	.
SPRLISCIII4977-16	NON-MUCOID	YES	Wze(Met79Arg);WchA(Ala454Pro)	.	T	.	-	C	.	.	.	.	.	.	G	C	.	.	.	.	.	.	.	.	.	.	.	.	.	C	.	.	.	.	.	.	.	.	.	.	.	C	.	.	.	.	.
SPRLISCIII4981-16	NON-MUCOID	YES	WchA(Ala321Asp)	.	T	.	-	C	.	.	.	.	.	.	.	C	.	.	.	.	.	.	.	.	A	.	.	.	.	.	.	.	.	.	.	.	.	.	.	.	.	C	.	.	.	.	.
SPRLISCIII4982-16	NON-MUCOID	YES	WchA truncated	.	T	.	-	C	.	.	.	.	.	.	.	C	.	.	.	.	.	.	.	.	.	.	.	.	.	.	.	.	.	.	.	.	.	.	.	.	.	C	.	.	.	.	.
SPRLISCIII5056-16	NON-MUCOID	YES	WchA(Arg346Cys)	.	T	.	-	C	.	.	.	.	.	.	.	C	.	.	.	.	.	.	.	.	.	T	.	.	.	.	.	.	.	.	.	.	.	.	.	.	.	C	.	.	.	.	.
SPRLISCIII5221-16	NON-MUCOID	YES	WchA truncated	.	T	.	-	C	.	.	.	.	.	.	.	C	.	.	.	.	.	.	.	.	.	.	.	.	.	.	.	.	.	.	.	.	.	.	.	.	.	C	.	.	.	.	.
SPRLISCIII5876-17	NON-MUCOID	NO		.	T	.	-	C	.	.	.	.	.	.	.	C	.	.	.	.	.	.	.	.	.	.	.	.	.	.	.	.	.	.	.	.	.	.	.	.	.	C	.	.	.	.	.
SPRLISCIII6025-18	NON-MUCOID	YES	WchA-Wci GAP	.	T	.	-	C	.	.	.	.	.	.	.	C	-	-	-	-	-	-	-	-	-	-	-	-	-	-	-	-	-	-	-	.	.	.	.	.	.	C	.	.	.	.	.
SPRLISCIII6158-18	NON-MUCOID	YES	WchA(deletion 121-131);WciS(Pro315Leu)	.	T	.	-	C	.	.	.	.	.	.	.	C	.	.	.	.	.	.	.	.	.	.	.	.	.	.	.	.	.	.	.	.	T	.	.	.	.	C	.	.	.	.	.
SPRLISCIII6253-18	NON-MUCOID	YES	WciR(Gly38Arg)	.	T	.	-	C	.	.	.	.	.	.	.	C	.	.	.	.	.	.	.	.	.	.	.	.	.	.	.	.	C	.	.	.	.	.	.	.	.	C	.	.	.	.	.
SPRLISCIII6273-18	NON-MUCOID	NO		.	T	.	-	C	.	.	.	.	.	.	.	C	.	.	.	.	.	.	.	.	.	.	.	.	.	.	.	.	.	.	.	.	.	.	.	.	.	C	.	.	.	.	.
SPRLISCIII6342-18	NON-MUCOID	YES	WchA(Val447Phe)	.	T	.	-	C	.	.	.	.	.	.	.	C	.	.	.	.	.	.	.	.	.	.	.	.	T	.	.	.	.	.	.	.	.	.	.	.	.	C	.	.	.	.	.
SPRLISCIII6347-18	NON-MUCOID	YES	WchA(Leu232Val)	.	T	.	-	C	.	.	.	.	.	.	.	C	.	.	.	.	G	.	.	.	.	.	.	.	.	.	.	.	.	.	.	.	.	.	.	.	.	C	.	.	.	.	.
SPRLISCIII6421-18	NON-MUCOID	NO		.	T	.	-	C	.	.	.	.	.	.	.	C	.	.	.	.	.	.	.	.	.	.	.	.	.	.	.	.	.	.	.	.	.	.	.	.	.	C	.	.	.	.	.
SPRLISCIII0136-20	MUCOID	NO		.	T	.	-	C	.	.	.	.	.	.	.	C	.	.	.	.	.	.	.	.	.	.	.	.	.	.	.	.	.	.	.	.	.	.	.	.	.	C	.	.	.	.	.
SPRLISCIII0210-20	MUCOID	NO		.	T	.	-	C	.	.	.	.	.	.	.	C	.	.	.	.	.	.	.	.	.	.	.	.	.	.	.	.	.	.	.	.	.	.	.	.	.	C	.	.	.	.	.
SPRLISCIII0296-20	MUCOID	NO		.	T	.	-	C	.	.	.	.	.	.	.	C	.	.	.	.	.	.	.	.	.	.	.	.	.	.	.	.	.	.	.	.	.	.	.	.	.	C	.	.	.	.	.
SPRLISCIII0342-20	MUCOID	NO		.	T	.	-	C	.	.	.	.	.	.	.	C	.	.	.	.	.	.	.	.	.	.	.	.	.	.	.	.	.	.	.	.	.	.	.	.	.	C	.	.	.	.	.
SPRLISCIII0345-20	MUCOID	NO		.	T	.	-	C	.	.	.	.	.	.	.	C	.	.	.	.	.	.	.	.	.	.	.	.	.	.	.	.	.	.	.	.	.	.	.	.	.	C	.	.	.	.	.
SPRLISCIII0387-20	MUCOID	NO		.	T	.	-	C	.	.	.	.	.	.	.	C	.	.	.	.	.	.	.	.	.	.	.	.	.	.	.	.	.	.	.	.	.	.	.	.	.	C	.	.	.	.	.
SPRLISCIII0433-20	MUCOID	NO		.	T	.	-	C	.	.	.	.	.	.	.	C	.	.	.	.	.	.	.	.	.	.	.	.	.	.	.	.	.	.	.	.	.	.	.	.	.	C	.	.	.	.	.
SPRLISCIII0474-20	MUCOID	NO		.	T	.	A	C	.	.	.	.	.	.	.	C	.	.	.	.	.	.	.	.	.	.	.	.	.	.	.	.	.	.	.	.	.	.	.	.	.	.	.	.	.	.	.
SPRLISCIII0696-20	MUCOID	NO		.	T	.	-	C	.	.	.	.	.	.	.	C	.	.	.	.	.	.	.	.	.	.	.	.	.	.	.	.	.	.	.	.	.	.	.	.	.	C	.	.	.	.	.
SPRLISCIII0849-08	MUCOID	NO		.	T	.	A	C	.	.	.	.	.	.	.	C	.	.	.	.	.	.	.	.	.	.	.	.	.	.	.	.	.	.	.	.	.	.	.	.	.	.	.	.	.	.	.
SPRLISCIII0973-08	MUCOID	NO		.	T	.	-	C	.	.	.	.	.	.	.	C	.	.	.	.	.	.	.	.	.	.	.	.	.	.	.	.	.	.	.	.	.	.	.	.	.	C	.	.	.	.	.
SPRLISCIII1088-08	MUCOID	NO		.	T	.	A	C	.	.	.	.	.	.	.	C	.	.	.	.	.	.	.	.	.	.	.	.	.	.	.	.	.	.	.	.	.	.	.	.	.	.	.	.	.	.	.
SPRLISCIII1383-09	MUCOID	NO		.	T	.	-	C	.	.	.	.	.	.	.	C	.	.	.	.	.	.	.	.	.	.	.	.	.	.	.	.	.	.	.	.	.	.	.	.	.	C	.	.	.	.	.
SPRLISCIII1592-09	MUCOID	NO		G	T	.	A	C	.	.	.	.	.	.	.	C	.	.	.	.	.	.	.	.	.	.	.	.	.	.	.	.	.	.	.	.	.	.	.	.	.	.	.	.	.	.	.
SPRLISCIII2060-09	MUCOID	NO		.	T	.	A	C	.	.	.	.	.	.	.	C	.	.	.	.	.	.	.	.	.	.	.	.	.	.	.	.	.	.	.	.	.	.	.	.	.	.	.	.	.	.	.
SPRLISCIII2085-09	MUCOID	NO		.	T	.	A	C	.	.	.	.	.	.	.	C	.	.	.	.	.	.	.	.	.	.	.	.	.	.	.	.	.	.	.	.	.	.	.	.	.	.	.	.	.	.	.
SPRLISCIII2339M1-10	MUCOID	NO		.	T	.	A	C	.	.	.	.	.	.	.	C	.	.	.	.	.	.	.	.	.	.	.	.	.	.	.	.	.	.	.	.	.	.	.	.	.	.	.	.	.	.	.
SPRLISCIII2409-10	MUCOID	NO		.	T	.	-	C	.	.	.	.	.	.	.	C	.	.	.	.	.	.	.	.	.	.	.	.	.	.	.	.	.	.	.	.	.	.	.	.	.	C	.	.	.	.	.
SPRLISCIII2746-11	MUCOID	NO		.	T	.	-	C	.	.	.	.	.	.	.	C	.	.	.	.	.	.	.	.	.	.	.	.	.	.	.	.	.	.	.	.	.	.	.	.	.	C	.	.	.	.	.
SPRLISCIII3021-11	MUCOID	NO		.	T	.	A	C	.	.	.	.	.	.	.	C	.	.	.	.	.	.	.	.	.	.	.	.	.	.	.	.	.	.	.	.	.	.	.	.	.	.	.	.	.	.	.
SPRLISCIII3128-11	MUCOID	NO		.	T	.	A	C	.	.	.	.	.	.	.	C	.	.	.	.	.	.	.	.	.	.	.	.	.	.	.	.	.	.	.	.	.	.	.	.	.	.	.	.	.	.	C
SPRLISCIII3193-12	MUCOID	NO		.	T	.	-	C	.	.	.	.	.	.	.	C	.	.	.	.	.	.	.	.	.	.	.	.	.	.	.	.	.	.	.	.	.	.	.	.	.	C	.	.	.	.	.
SPRLISCIII3372-12	MUCOID	NO		.	T	.	-	C	.	.	.	.	.	.	.	C	.	.	.	.	.	.	.	.	.	.	.	.	.	.	.	.	.	.	.	.	.	.	.	.	.	C	.	.	.	.	.
SPRLISCIII3421-12	MUCOID	NO		.	T	.	A	C	.	.	.	.	.	.	.	C	.	.	.	.	.	.	.	.	.	.	.	.	.	.	.	.	.	.	.	.	.	.	.	.	.	.	.	.	.	.	.
SPRLISCIII3540-12	MUCOID	NO		.	T	.	A	C	.	.	.	.	.	.	.	C	.	.	.	.	.	.	.	.	.	.	.	.	.	.	.	.	.	.	.	.	.	.	.	.	.	.	.	.	.	.	.
SPRLISCIII3610-13	MUCOID	NO		.	T	.	-	C	.	.	.	.	.	.	.	C	.	.	.	.	.	.	.	.	.	.	.	.	.	.	.	.	.	.	.	.	.	.	.	.	.	C	.	.	.	.	.
SPRLISCIII3776-13	MUCOID	NO		.	T	.	-	C	.	.	.	.	.	.	.	C	.	.	.	.	.	.	.	.	.	.	.	.	.	.	.	.	.	.	.	.	.	.	.	.	.	C	.	.	.	.	.
SPRLISCIII3832-13	MUCOID	NO		.	T	.	A	C	.	.	.	.	.	.	.	C	.	.	.	.	.	.	.	.	.	.	.	.	.	.	.	.	.	.	.	.	.	.	.	.	.	.	.	.	.	.	.
SPRLISCIII3859-13	MUCOID	NO		.	T	.	-	C	.	.	.	.	.	.	.	C	.	.	.	.	.	.	.	.	.	.	.	.	.	.	.	.	.	.	.	.	.	.	.	.	.	C	.	.	.	.	.
SPRLISCIII4063-14	MUCOID	NO		.	T	.	-	C	.	.	.	.	.	.	.	C	.	.	.	.	.	.	.	.	.	.	.	.	.	.	.	.	.	.	.	.	.	.	.	.	.	C	.	.	.	.	.
SPRLISCIII4071-14	MUCOID	NO		.	T	.	A	C	.	.	.	.	.	.	.	C	.	.	.	.	.	.	.	.	.	.	.	.	.	.	.	.	.	.	.	.	.	.	.	.	.	.	.	.	.	.	.
SPRLISCIII4247-14	MUCOID	NO		.	T	.	-	C	.	.	.	.	.	.	.	C	.	.	.	.	.	.	.	.	.	.	.	.	.	.	.	.	.	.	.	.	.	.	.	.	.	C	.	.	.	.	.
SPRLISCIII4285-14	MUCOID	NO		.	.	A	-	C	.	.	.	.	.	.	.	.	.	.	.	.	.	.	.	.	.	.	.	.	.	.	.	.	.	.	.	.	.	G	.	.	.	.	G	.	.	.	.
SPRLISCIII4411-15	MUCOID	NO		.	T	.	-	C	.	.	.	.	.	.	.	C	.	.	.	.	.	.	.	.	.	.	.	.	.	.	.	.	.	.	.	.	.	.	.	.	.	C	.	.	.	.	.
SPRLISCIII4673-15	MUCOID	NO		.	T	.	-	C	.	.	.	.	.	.	.	C	.	.	.	.	.	.	.	.	.	.	.	.	.	.	.	.	.	.	.	.	.	.	.	.	.	C	.	.	.	.	.
SPRLISCIII4791-15	MUCOID	NO		.	T	.	-	C	.	.	.	.	.	.	.	C	.	.	.	.	.	.	.	.	.	.	.	.	.	.	.	.	.	.	.	.	.	.	.	.	.	C	.	.	.	.	.
SPRLISCIII5029-16	MUCOID	NO		.	T	.	-	C	.	.	.	.	.	.	.	C	.	.	.	.	.	.	.	.	.	.	.	.	.	.	.	.	.	.	.	.	.	.	.	.	.	C	.	.	.	.	.
SPRLISCIII5031-16	MUCOID	YES	WciS(Ala99Val)	.	T	.	A	C	.	.	.	.	.	.	.	C	.	.	.	.	.	.	.	.	.	.	.	.	.	.	.	.	.	.	T	.	.	.	.	.	.	.	.	.	.	.	.
SPRLISCIII5078-16	MUCOID	NO		.	T	.	-	C	.	.	.	.	.	.	.	C	.	.	.	.	.	.	.	.	.	.	.	.	.	.	.	.	.	.	.	.	.	.	.	.	.	C	.	.	.	.	.
SPRLISCIII5150-16	MUCOID	NO		.	T	.	-	C	.	.	.	.	.	.	.	C	.	.	.	.	.	.	.	.	.	.	.	.	.	.	.	.	.	.	.	.	.	.	.	.	.	C	.	.	.	.	.
SPRLISCIII5174-16	MUCOID	NO		.	.	A	-	C	.	.	.	.	.	.	.	.	.	.	.	.	.	.	.	.	.	.	.	.	.	.	.	.	.	.	.	.	.	G	.	.	.	.	G	.	.	.	.
SPRLISCIII5178-16	MUCOID	NO		.	.	A	-	C	.	.	.	.	.	.	.	.	.	.	.	.	.	.	.	.	.	.	.	.	.	.	.	.	.	.	.	.	.	.	.	.	.	.	.	.	.	.	.
SPRLISCIII5221M1-16	MUCOID	NO		.	T	.	-	C	.	.	.	.	.	.	.	C	.	.	.	.	.	.	.	.	.	.	.	.	.	.	.	.	.	.	.	.	.	.	.	.	.	C	.	.	.	.	.
SPRLISCIII5232-16	MUCOID	NO		.	T	.	-	C	.	.	.	.	.	.	.	C	.	.	.	.	.	.	.	.	.	.	.	.	.	.	.	.	.	.	.	.	.	.	.	.	.	C	.	.	.	.	.
SPRLISCIII5272-16	MUCOID	YES	Wze(Met36Ile)	.	T	.	-	C	.	.	.	T	.	.	.	C	.	.	.	.	.	.	.	.	.	.	.	.	.	.	.	.	.	.	.	.	.	.	.	.	.	C	.	.	.	.	.
SPRLISCIII5345-16	MUCOID	NO		.	T	.	-	C	.	.	.	.	.	.	.	C	.	.	.	.	.	.	.	.	.	.	.	.	.	.	.	.	.	.	.	.	.	.	.	.	.	C	.	.	.	.	.
SPRLISCIII5354-16	MUCOID	NO		.	T	.	A	C	.	.	.	.	.	.	.	C	.	.	.	.	.	.	.	.	.	.	.	.	.	.	.	.	.	.	.	.	.	.	.	.	.	.	.	.	.	.	.
SPRLISCIII5371-17	MUCOID	NO		.	T	.	-	C	.	.	.	.	.	.	.	C	.	.	.	.	.	.	.	.	.	.	.	.	.	.	.	.	.	.	.	.	.	.	.	.	.	C	.	.	.	.	.
SPRLISCIII5409-17	MUCOID	NO		.	T	.	A	C	.	.	.	.	.	.	.	C	.	.	.	.	.	.	.	.	.	.	.	.	.	.	.	.	.	.	.	.	.	.	.	.	.	.	.	.	.	.	.
SPRLISCIII5422-17	MUCOID	NO		.	T	.	-	C	.	.	.	.	.	.	.	C	.	.	.	.	.	.	.	.	.	.	.	.	.	.	.	.	.	.	.	.	.	.	.	.	.	C	.	.	.	.	.
SPRLISCIII5462-17	MUCOID	NO		.	T	.	-	C	.	.	.	.	.	.	.	C	.	.	.	.	.	.	.	.	.	.	.	.	.	.	.	.	.	.	.	.	.	.	.	.	.	C	.	.	.	.	.
SPRLISCIII5464-17	MUCOID	NO		.	T	.	-	C	.	.	.	.	.	.	.	C	.	.	.	.	.	.	.	.	.	.	.	.	.	.	.	.	.	.	.	.	.	.	.	.	.	C	.	.	.	.	.
SPRLISCIII5469-17	MUCOID	YES	Wze(Tyr65Phe)	.	T	.	-	C	.	.	.	.	.	T	.	C	.	.	.	.	.	.	.	.	.	.	.	.	.	.	.	.	.	.	.	.	.	.	.	.	.	C	.	.	.	.	.
SPRLISCIII5491-17	MUCOID	NO		.	.	A	-	C	.	.	.	.	.	.	.	.	.	.	.	.	.	.	.	.	.	.	.	.	.	.	.	.	.	.	.	.	.	G	.	.	.	.	G	.	.	.	.
SPRLISCIII5876M1-17	MUCOID	NO		.	T	.	-	C	.	.	.	.	.	.	.	C	.	.	.	.	.	.	.	.	.	.	.	.	.	.	.	.	.	.	.	.	.	.	.	.	.	C	.	.	.	.	.
SPRLISCIII5876M2-17	MUCOID	NO		.	T	.	-	C	.	.	.	.	.	.	.	C	.	.	.	.	.	.	.	.	.	.	.	.	.	.	.	.	.	.	.	.	.	.	.	.	.	C	.	.	.	.	.
SPRLISCIII5919-17	MUCOID	NO		.	T	.	-	C	.	.	.	.	.	.	.	C	.	.	.	.	.	.	.	.	.	.	.	.	.	.	.	.	.	.	.	.	.	.	.	.	.	C	.	.	.	.	.
SPRLISCIII5945-18	MUCOID	NO		.	T	.	A	C	.	.	.	.	.	.	.	C	.	.	.	.	.	.	.	.	.	.	.	.	.	.	.	.	.	.	.	.	.	.	.	.	.	.	.	.	.	.	.
SPRLISCIII5995-18	MUCOID	NO		.	T	.	A	C	.	.	.	.	.	.	.	C	.	.	.	.	.	.	.	.	.	.	.	.	.	.	.	.	.	.	.	.	.	.	.	.	.	.	.	.	.	.	.
SPRLISCIII6003-18	MUCOID	NO		.	T	.	-	C	.	.	.	.	.	.	.	C	.	.	.	.	.	.	.	.	.	.	.	.	.	.	.	.	.	.	.	.	.	.	.	.	.	C	.	.	.	.	.
SPRLISCIII6011-18	MUCOID	NO		.	T	.	A	C	.	.	.	.	.	.	.	C	.	.	.	.	.	.	.	.	.	.	.	.	.	.	.	.	.	.	.	.	.	.	.	.	.	.	.	.	.	.	.
SPRLISCIII6014-18	MUCOID	NO		.	T	.	-	C	.	.	.	.	.	.	.	C	.	.	.	.	.	.	.	.	.	.	.	.	.	.	.	.	.	.	.	.	.	.	.	.	.	C	.	.	.	.	.
SPRLISCIII6025M1-18	MUCOID	NO		.	T	.	-	C	.	.	.	.	.	.	.	C	.	.	.	.	.	.	.	.	.	.	.	.	.	.	.	.	.	.	.	.	.	.	.	.	.	C	.	.	.	.	.
SPRLISCIII6085-18	MUCOID	NO		.	T	.	A	C	.	.	.	.	.	.	.	C	.	.	.	.	.	.	.	.	.	.	.	.	.	.	.	.	.	.	.	.	.	.	.	.	.	.	.	.	.	.	.
SPRLISCIII6158M1-18	MUCOID	NO		.	T	.	-	C	.	.	.	.	.	.	.	C	.	.	.	.	.	.	.	.	.	.	.	.	.	.	.	.	.	.	.	.	.	.	.	.	.	C	.	.	.	.	.
SPRLISCIII6253M1-18	MUCOID	NO		.	T	.	-	C	.	.	.	.	.	.	.	C	.	.	.	.	.	.	.	.	.	.	.	.	.	.	.	.	.	.	.	.	.	.	.	.	.	C	.	.	.	.	.
SPRLISCIII6304-18	MUCOID	NO		.	T	.	-	C	.	.	.	.	.	.	.	C	.	.	.	.	.	.	.	.	.	.	.	.	.	.	.	.	.	.	.	.	.	.	.	.	.	C	.	.	.	.	.
SPRLISCIII6498-18	MUCOID	NO		.	T	.	A	C	.	.	.	.	.	.	.	C	.	.	.	.	.	.	.	.	.	.	.	.	.	.	.	.	.	.	.	.	.	.	.	.	.	.	.	.	.	.	.
SPRLISCIII0269-20	INTERMEDIATE	YES	Upg(His309Asp)	.	.	A	-	C	.	.	.	.	.	.	.	.	.	.	.	.	.	.	.	.	.	.	.	.	.	.	.	.	.	.	.	.	.	G	.	.	.	.	.	.	.	.	.
SPRLISCIII0785-08	INTERMEDIATE	YES	Wzd(Val124Ile); Wze(Met36Ile);Wzx(Lys279Tyr-Asn-Glu-Ala-Lys)	.	T	.	A	C	.	A	.	A	.	.	.	C	.	.	.	.	.	.	.	.	.	.	.	.	.	.	.	.	.	.	.	.	.	.	.	.	.	.	.	.	.	.	.
SPRLISCIII1229-08	INTERMEDIATE	NO		.	T	.	A	C	.	.	.	.	.	.	.	C	.	.	.	.	.	.	.	.	.	.	.	.	.	.	.	.	.	.	.	.	.	.	.	.	.	.	.	.	.	.	.
SPRLISCIII1540-09	INTERMEDIATE	NO		.	T	.	A	C	.	.	.	.	.	.	.	C	.	.	.	.	.	.	.	.	.	.	.	.	.	.	.	.	.	.	.	.	.	.	.	.	.	.	.	.	.	.	.
SPRLISCIII1555-09	INTERMEDIATE	NO		.	T	.	A	C	.	.	.	.	.	.	.	C	.	.	.	.	.	.	.	.	.	.	.	.	.	.	.	.	.	.	.	.	.	.	.	.	.	.	.	.	.	.	.
SPRLISCIII2302-10	INTERMEDIATE	NO		.	T	.	A	C	.	.	.	.	.	.	.	C	.	.	.	.	.	.	.	.	.	.	.	.	.	.	.	.	.	.	.	.	.	.	.	.	.	.	.	.	.	.	.

SPRLISCIII3095-19	INTERMEDIATE	YES	WciS(His289Gln)	.	T	.	-	C	.	.	.	.	.	.	.	C	.	.	.	.	.	.	.	.	.	.	.	.	.	.	.	.	.	.	.	A	.	.	.	.	.	C	.	.	.	.	.
SPRLISCIII3371-19	INTERMEDIATE	YES	Upg(Ala386Pro)	.	T	.	-	C	.	.	.	.	.	.	.	C	.	.	.	.	.	.	.	.	.	.	.	.	.	.	.	.	.	.	.	.	.	.	.	.	.	C	.	.	.	.	.
SPRLISCIII4054-14	INTERMEDIATE	YES	Wze(Met36Ile);(WchAGly269Ser)	.	T	.	-	C	.	.	.	A	.	.	.	C	.	.	.	.	.	.	A	.	.	.	.	.	.	.	.	.	.	.	.	.	.	.	.	.	.	C	.	.	.	.	.
SPRLISCIII4421-15	INTERMEDIATE	YES	Wzx(Leu268Val)	.	T	.	A	C	.	.	.	.	.	.	.	C	.	.	.	.	.	.	.	.	.	.	.	.	.	.	.	.	.	.	.	.	.	.	.	G	.	.	.	.	.	.	.
SPRLISCIII4981M1-16	INTERMEDIATE	YES	Wzx(Pro269Ser)	.	T	.	-	C	.	.	.	.	.	.	.	C	.	.	.	.	.	.	.	.	.	.	.	.	.	.	.	.	.	.	.	.	.	.	.	.	T	C	.	.	G	.	.
SPRLISCIII4982M1-16	INTERMEDIATE	YES	Upg(Pro275Arg)	.	T	.	-	C	.	.	.	.	.	.	.	C	.	.	.	.	.	.	.	.	.	.	.	.	.	.	.	.	.	.	.	.	.	.	.	.	.	C	.	.	.	.	.
SPRLISCIII5027-16	INTERMEDIATE	YES	WchA(Asp229Glu)	.	T	.	-	C	.	.	.	.	.	.	.	C	.	.	.	A	.	.	.	.	.	.	.	.	.	.	.	.	.	.	.	.	.	.	.	.	.	C	.	.	.	.	.
SPRLISCIII5038-16	INTERMEDIATE	YES	WciQ truncated	.	T	.	-	C	.	.	.	.	.	.	.	C	.	.	.	.	.	.	.	.	.	.	.	.	.	.	.	.	.	.	.	.	.	.	.	.	.	C	.	.	.	.	.
SPRLISCIII5517-17	INTERMEDIATE	YES	Wzd(Gln62Arg);WchA(Leu210Arg)	.	T	.	-	C	G	.	.	.	.	.	.	C	.	.	G	.	.	.	.	.	.	.	.	.	.	.	.	.	.	.	.	.	.	.	.	.	.	C	.	.	.	.	.
SPRLISCIII5796-17	INTERMEDIATE	YES	WciQ truncated	.	T	.	-	C	.	.	.	.	.	.	.	C	.	.	.	.	.	.	.	.	.	.	.	.	.	.	.	.	.	.	.	.	.	.	.	.	.	C	.	.	.	.	.
SPRLISCIII6005-18	INTERMEDIATE	NO		.	T	.	A	C	.	.	.	.	.	.	.	C	.	.	.	.	.	.	.	.	.	.	.	.	.	.	.	.	.	.	.	.	.	.	.	.	.	.	.	.	.	.	.
SPRLISCIII6322-18	INTERMEDIATE	YES	WciQ truncated	.	T	.	-	C	.	.	.	.	.	.	.	C	.	.	.	.	.	.	.	.	.	.	.	.	.	.	.	.	.	.	.	.	.	.	.	.	.	C	.	.	.	.	.
SPRLISCIII6330-18	INTERMEDIATE	NO		.	T	.	-	C	.	.	G	.	.	.	.	C	.	.	.	.	.	.	.	.	.	.	.	.	.	.	.	.	.	C	.	.	.	.	.	.	.	C	.	T	.	.	.
SPRLISCIII6345-18	INTERMEDIATE	YES	Upg(Leu204Phe)	.	T	.	-	C	.	.	.	.	.	.	.	C	.	.	.	.	.	.	.	.	.	.	.	.	.	.	.	.	.	.	.	.	.	.	.	.	.	C	.	.	.	.	.
